# Natural bioactive products in the regulation of bone metabolism and regeneration

**DOI:** 10.3389/fphar.2025.1683279

**Published:** 2025-11-14

**Authors:** Xinyi Ouyang, Qiang Ma, Chang Zhou, Jiaqian Tang, Mengyuan Li, Jun Qing, Xiaoming Lei, Dan Huang, Huiping Liu, Guomin Zhang

**Affiliations:** 1 Hunan University of Chinese Medicine, Changsha, China; 2 The Key Laboratory of Hunan Province for Integrated Traditional Chinese and Western Medicine on Prevention and Treatment of Cardio-Cerebral Diseases, College of Integrated Traditional Chinese and Western Medicine, Hunan University of Chinese Medicine, Changsha, China; 3 Hunan Key Laboratory of Traditional Chinese Medicine Prescription and Syndromes Translational Medicine, Hunan University of Chinese Medicine, Changsha, China; 4 State Key Laboratory of Chinese Medicine Powder and Medicine Innovation in Hunan (Incubation), Science and Technology Innovation Center, Hunan University of Chinese Medicine, Changsha, China

**Keywords:** natural bioactive products, osteoporosis, bone regeneration, osteo-angiogenic coupling, immune modulation, ferroptosis, gut microbiota, Gut-bone axis

## Abstract

Osteoporosis (OP) is a systemic skeletal disorder characterized by decreased bone mineral density (BMD), impaired bone microarchitecture, and an elevated risk of fragility fractures. Although conventional pharmacological agents—such as bisphosphonates, selective estrogen receptor modulators, and monoclonal antibodies—can attenuate disease progression, their long-term application is limited by adverse effects and suboptimal patient adherence. Consequently, there is growing interest in the development of safer, multi-targeted therapeutic strategies. Plant-derived bioactive products have garnered increasing attention due to their broad pharmacological profiles, including the promotion of osteoblastogenesis, suppression of osteoclastogenesis, regulation of bone–vascular coupling, and modulation of immune and oxidative stress pathways. Recent advances in biomaterial-assisted delivery systems have further improved the physicochemical stability, bioavailability, and tissue-specific delivery of these phytochemicals, thereby enhancing their therapeutic efficacy in bone regeneration. Although accumulated *in vitro* and *in vivo* studies suggest the bone-protective potential of these natural agents, clinical translation remains limited. Further mechanistic investigations and rigorously designed clinical trials are warranted to substantiate their efficacy and safety in human populations. This review summarizes recent progress in the mechanistic understanding of natural products involved in bone metabolism, with a particular focus on representative classes such as flavonoids, polyphenols, polysaccharides, glycosides, and terpenoids. In addition, we discuss the translational potential of integrating these agents with advanced drug delivery platforms, aiming to provide a theoretical framework and future research directions for the treatment of OP and related bone disorders.

## 1 Introduction

OP is a prevalent systemic metabolic bone disorder that primarily affects the elderly and postmenopausal women. It is characterized by reduced BMD and compromised bone microarchitecture, ultimately leading to increased skeletal fragility and a higher incidence of fragility fractures (Mizoguchi and Ono, 2021; Ponzetti and Rucci, 2021).

With the rapid global increase in aging populations, the prevalence of OP is escalating, positioning it as a major public health concern with profound implications for morbidity, mortality, and healthcare systems worldwide. In particular, osteoporotic fractures result in significant pain, functional impairment, and loss of independence, imposing substantial socioeconomic and psychological burdens on patients and caregivers (Arceo-Mendoza and Camacho, 2021; Kanis et al., 2004).

Current therapeutic strategies for OP primarily include antiresorptive agents (e.g., bisphosphonates), anabolic drugs (e.g., parathyroid hormone analogs), hormone replacement therapy, and supplementation with calcium and vitamin D. Among these, oral bisphosphonates and parathyroid hormone analogs are the most commonly prescribed (Ayers et al., 2023; Eastell, 1998; Ensrud and Crandall, 2024). However, these pharmacological interventions are frequently associated with adverse events, such as gastrointestinal irritation, esophagitis, and esophageal ulcers. Moreover, the discontinuation of these treatments may lead to a rebound in bone turnover and an increased risk of fractures, sometimes exceeding baseline levels (Kanis et al., 2019; Sk and Bz, 2024; Stupski et al., 2021).

Consequently, there is a critical need to develop safer and more effective therapeutic alternatives with fewer side effects and sustained long-term benefits. Recent studies have increasingly focused on natural products and multi-targeted interventions as promising avenues for future OP management (Lei et al., 2023; Zhivodernikov et al., 2023).

The pathogenesis of OP is multifactorial and involves both intrinsic and extrinsic contributors, including aging, estrogen deficiency, endocrine dysfunction, chronic inflammation, oxidative stress, and diminished mechanical loading (Manolagas, 2010). These factors collectively disrupt bone homeostasis by promoting bone resorption and/or impairing bone formation, ultimately leading to reduced BMD, degradation of bone microarchitecture, and increased fracture susceptibility.

In addition to the aforementioned mechanisms, a wide spectrum of lifestyle and systemic factors also contribute to the development and progression of OP. These include nutritional deficiencies (e.g., inadequate calcium and vitamin D intake), genetic predisposition, smoking, excessive alcohol consumption, prolonged glucocorticoid therapy, and physical inactivity. Moreover, endocrine disorders such as hyperthyroidism and diabetes, as well as chronic inflammatory diseases like rheumatoid arthritis, are well-established secondary causes of OP (Laurent et al., 2022; Ratajczak et al., 2021; Yang et al., 2021). These diverse factors act synergistically to impair bone remodeling and accelerate bone loss. A comprehensive understanding of their complex interplay is essential for the development of effective, individualized prevention and treatment strategies. In this context, the integration of phytochemicals as adjunctive agents within broader therapeutic regimens may offer additional benefits.

Under physiological conditions, bone is a highly dynamic tissue that undergoes continuous remodeling to maintain structural integrity and mineral homeostasis (Kenkre and Bassett, 2018). Beyond its mechanical role during growth and locomotion, the skeleton undergoes lifelong renewal regulated by the tightly coordinated activity of osteoblasts and osteoclasts (Chen et al., 2012). Osteoblasts—the primary bone-forming cells—synthesize and secrete extracellular matrix (ECM) proteins and facilitate matrix mineralization, thereby maintaining bone strength and density. They also play a pivotal role in skeletal repair, structural maintenance, and the regulation of bone metabolism (Stein et al., 1996).

When bone resorption persistently exceeds bone formation, the delicate balance between osteoblasts and osteoclasts is disrupted. This imbalance leads to progressive bone loss, deterioration of bone microarchitecture, and elevated fracture risk, thereby contributing to the pathogenesis of various metabolic bone disorders, particularly OP (Seeman and Delmas, 2006). Against this backdrop, bone regeneration has garnered increasing attention as a fundamental physiological process essential for maintaining skeletal homeostasis and repairing structural damage. It has consequently become a central focus in both basic and translational research.

Bone regeneration refers to the highly orchestrated biological process by which bone structure is restored and functional integrity is reestablished following injury, structural defect, or metabolic dysregulation. This process involves the coordinated interplay among osteoblasts, mesenchymal stem cells, endothelial cells, and products of the bone microenvironment (Henkel et al., 2013). In addition to its critical role in fracture healing and defect repair, bone regeneration represents a core mechanism underlying the therapeutic response in metabolic bone diseases. Therefore, elucidating the cellular and molecular mechanisms that regulate bone regeneration is of paramount importance for the development of novel interventions for OP and related skeletal disorders.

In recent years, natural bioactive products—particularly those derived from traditional botanical drug—have emerged as promising candidates for modulating bone remodeling due to their favorable biocompatibility, low toxicity, and pleiotropic pharmacological activities (Que et al., 2022; Xi et al., 2022). A growing body of evidence suggests that these phytochemicals exert multi-targeted regulatory effects on bone regeneration by modulating key signaling pathways, including Wingless-related integration site (Wnt)/β-catenin, Bone morphogenetic protein (BMP)/mothers against decapentaplegic homolog (Smad), Phosphoinositide 3-kinase (PI3K)/protein kinase B (Akt), and Nuclear factor erythroid 2–related factor 2 (Nrf2)/heme oxygenase-1 (HO-1). These products have been shown to enhance osteoblast differentiation and function, promote angiogenesis, suppress oxidative stress and inflammation, and inhibit osteoclastogenesis—collectively contributing to an improved bone microenvironment and exerting comprehensive osteoprotective effects.

Building upon these mechanistic insights, this review aims to provide a systematic overview of recent advances in the application of natural bioactive products for OP therapy, with a particular emphasis on their roles in promoting bone regeneration. Key areas of discussion include the coordinated regulation of osteogenesis and angiogenesis, modulation of the bone immune microenvironment, mitigation of oxidative damage, and enhancement of bone repair through integration with advanced drug delivery platforms.

Through multi-pathway and multi-target modulation, natural products hold considerable promise in restoring bone tissue homeostasis, enhancing regenerative capacity, and expanding their applicability in bone tissue engineering and regenerative medicine. These findings collectively provide a theoretical framework and technical basis for the development of safer, more effective, and clinically translatable therapeutic strategies for osteoporosis.

## 2 Methods

This review was conducted through a comprehensive literature search using the PubMed database. The following keywords and MeSH terms were employed—individually or in combination—to identify relevant studies: “natural bioactive products,” “flavonoids,” “phenolics,” “saponins,” “polysaccharides,” “terpenoids,” “osteogenesis,” “bone regeneration,” “osteoporosis,” “osteoblasts,” “osteoclasts,” “angiogenesis,” “bone marrow-derived mesenchymal stem cells (BMSCs),” “Wnt/β-catenin,” “BMP/Smad,” “phosphatidylinositol 3-kinase/protein kinase B (PI3K/Akt),” “Mitogen-activated protein kinase (MAPK),” “Nuclear factor kappa-light-chain-enhancer of activated B cells (NF-κB),” “Janus kinase (JAK)/signal transducer and activator of transcription (STAT),” “Nuclear factor erythroid 2–related factor 2 (Nrf2)/glutathione peroxidase 4 (GPX4),” “ferroptosis,” “oxidative stress,” “immune modulation,” “Classically activated macrophage (M1)/alternatively activated macrophage (M2) polarization,” “regulatory T cells (Tregs),” “gut microbiota,” “gut–bone axis,” “Receptor activator of nuclear factor κB ligand (RANKL)/osteoprotegerin (OPG),” “drug delivery,” and “biomaterial scaffolds.” Boolean operators (AND, OR) were used to optimize search sensitivity and specificity.

Studies were included based on the following criteria:

1. Mechanistic studies *in vitro*: Articles investigating cellular and molecular mechanisms of natural bioactive products, with an emphasis on their regulatory roles in key signaling pathways relevant to the bone microenvironment (e.g., Wnt/β-catenin, PI3K/Akt, MAPK, Nrf2/HO-1, NF-κB).
2. Preclinical *in vivo* studies: Experimental studies involving animal models of bone-related disorders (e.g., osteoporosis, bone defects, or fracture healing), particularly those exploring therapeutic efficacy and translational relevance.

To ensure a broad and representative overview, studies involving diverse plant sources and product classes were considered. Priority was given to publications with well-defined experimental design, clear mechanistic interpretation, and higher levels of evidence. Given the current limitations in clinical translation, most of the included studies were based on *in vitro* and animal models rather than large-scale clinical trials. Publications from 2010 to 2025 were considered, with emphasis on recently published articles (2020–2025) to ensure that the review reflects the latest mechanistic and translational advances.

## 3 Natural products targeting osteogenic and osteoclastic signaling pathways

Traditional Chinese medicine (TCM) offers distinct advantages in the prevention and treatment of osteoporosis, primarily owing to the multi-target regulatory effects of its diverse natural bioactive constituents on bone regeneration. These products—commonly derived from traditional botanical drug and classified into flavonoids, glycosides, phenolics, polysaccharides, steroids, and terpenoids—have been shown to modulate osteoblast activity, activate key osteogenic signaling cascades, and improve the bone regenerative microenvironment. Through these mechanisms, they contribute to both structural reconstruction and functional restoration of bone tissue, thereby alleviating OP-associated bone loss and fragility, and exhibiting promising therapeutic potential for bone protection and repair.

At the core of bone regeneration lies the tightly regulated balance between bone formation and resorption, with osteoblast function serving as a principal determinant of bone-forming capacity (Raggatt and Partridge, 2010). Several canonical signaling pathways—including BMPs, Wnt/β-catenin, mitogen-activated protein kinases, and Notch—have been identified as critical regulators of osteogenic differentiation (Wagley et al., 2020; Wu et al., 2016). In parallel, transcription factors such as runt-related *transcription factor 2 (Runx2*) and *Osterix* orchestrate the gene expression programs necessary for osteoblast lineage commitment and functional maturation (Zhu et al., 2024). Moreover, extrinsic factors—including reactive oxygen species (ROS), pro-inflammatory cytokines, and hormonal fluctuations—exert additional influence by modulating the local bone microenvironment, thereby impacting the osteogenic process (Iantomasi et al., 2023).

Natural bioactive products derived from TCM can modulate these critical regulatory pathways at multiple levels, thereby facilitating osteogenic differentiation and enhancing bone formation. Notably, many of these products retain their regenerative efficacy even under pathological conditions such as skeletal injury, metabolic dysregulation, or inflammatory stress, which aligns closely with the current paradigms in bone biology and regenerative medicine.

In the following sections, representative products from distinct chemical classes will be systematically reviewed to elucidate their molecular mechanisms in promoting bone regeneration. Emphasis will be placed on their interactions with key signaling cascades, transcriptional regulators, and components of the bone microenvironment that collectively govern osteogenesis and skeletal repair.

### 3.1 Promotion of osteogenic differentiation via Wnt/β-catenin and BMPs signaling

The Wnt/β-catenin and BMPs signaling pathways are two fundamental regulatory axes in osteogenesis. These pathways directly influence osteoblast differentiation, matrix protein expression, and the overall rate and quality of bone formation (Day et al., 2005).

Activation of the canonical Wnt/β-catenin pathway is initiated by the binding of Wnt ligands to the Frizzled and Low-density lipoprotein receptor-related protein 5/6 (LRP5/6) receptor complex, which inhibits the phosphorylation and proteasomal degradation of β-catenin. Stabilized β-catenin subsequently translocates to the nucleus, where it interacts with T-cell factor/lymphoid enhancer-binding factor (TCF/LEF) transcription factors to activate osteogenic target genes such as *Runx2*, *ALP*, and *osteocalcin (OCN)* (Gaur et al., 2005).

The BMP signaling cascade is triggered by the binding of BMP-2 or BMP-4 to BMP type I and type II serine/threonine kinase receptors, resulting in the phosphorylation of Mothers against decapentaplegic homolog 1/5/8 (Smad1/5/8). These phosphorylated Smads form a heteromeric complex with Smad4, which translocates to the nucleus to upregulate *Runx2* and *Osterix*, thereby promoting osteoblast differentiation and matrix mineralization (Phimphilai et al., 2006). Together, the Wnt and BMP pathways constitute core regulators of bone regeneration, acting through both transcriptional and post-transcriptional mechanisms.

Numerous natural flavonoids have been shown to activate these osteoinductive pathways. For example, Albiflorin, a monoterpene glycoside isolated from the roots of *Paeonia lactiflora* Pall. (Paeoniaceae), enhances *Runx2* expression and osteogenic differentiation in Mouse calvaria-derived pre-osteoblast cell line (MC3T3-E1) osteoblasts by co-activating the Wnt/β-catenin and BMP signaling pathways. *In vivo*, it significantly accelerates fracture healing and bone mineralization in a rat femoral defect model (Kim et al., 2021). Genistein, an isoflavone isolated from the seeds of *Glycine* max (L.) Merr. (Fabaceae), has also been reported to promote the osteogenic differentiation of human bone marrow-derived mesenchymal stem cells (hBMSCs) by activating the BMP2/Smad5/Runx2 axis. Notably, this effect is abrogated by treatment with Noggin—a BMP antagonist—or by *Smad5* gene silencing, confirming the pathway’s involvement (Dai et al., 2013).

Recent studies have highlighted the osteoinductive potential of natural polysaccharides through multi-pathway regulation. A bioactive compound isolated from Curculigo orchioides (COP70-1), promotes osteogenesis by co-activating BMP2/Smad and Wnt/β-catenin pathways, upregulating *RUNX2*, *Osterix*, and related markers. Inhibitor studies confirm its dual-pathway mechanism in enhancing osteoblast differentiation. (Wang J et al., 2023). Polysaccharides activate BMP2/Smad signaling to increase *Collagen type I alpha 1 chain (COL1A1)*, *Alkaline phosphatase (ALP)*, and *Osteopontin (OPN)* expression, while suppressing Peroxisome proliferator-activated receptor gamma (PPARγ) to inhibit adipogenesis, thereby promoting Adipose-derived mesenchymal stem cell (ADSC) osteogenic differentiation and improving Ovariectomy (OVX)-induced osteoporosis (Yao et al., 2025). These findings underscore the multi-target regulatory capacity of polysaccharides and support their potential as therapeutic agents for OP.

Other flavonoids, including baicalein, a flavone isolated from the roots of *Scutellaria baicalensis* Georgi (Lamiaceae); apigenin, a flavone derived from the leaves and stems of *Apium graveolens* L. (Apiaceae); and quercetin, a flavonol widely distributed in the flowers of *Sophora japonica* L. (Fabaceae), the bulbs of *Allium cepa* L. (Amaryllidaceae), and the leaves of *Camellia sinensis* (L.) Kuntze (Theaceae), have been shown to enhance Wnt signaling through various mechanisms. These include stabilizing β-catenin, promoting its nuclear translocation, upregulating Frizzled and LRP5/6 expression, and inhibiting glycogen synthase kinase-3β (GSK-3β) activity. These actions collectively enhance the expression of osteogenic markers such as *ALP*, *Collagen type I (COL1)*, and *OCN in vitro*, contributing to their pro-osteogenic effects (Bian et al., 2021; Guo et al., 2011; Pan et al., 2021).

In summary, the Wnt/β-catenin and BMP signaling pathways play pivotal roles in regulating osteoblast differentiation and bone formation. The ability of flavonoids to target and activate these pathways highlights their mechanistic relevance and therapeutic potential in the treatment of osteoporosis.

### 3.2 Enhancement of matrix formation and osteoblast survival via PI3K/Akt, MAPK and apoptosis signaling

During bone formation, osteoblasts are not only responsible for synthesizing and mineralizing the ECM but must also maintain cellular viability to sustain long-term bone remodeling. Among the signaling pathways involved, PI3K/Akt and MAPK pathways play essential roles in regulating osteoblast differentiation and matrix production.

Activation of the PI3K/Akt pathway upregulates the expression of key osteogenic genes such as *Runx2*, *ALP*, and *COL1*, and promotes the synthesis of bone matrix proteins, thereby enhancing osteoblastic function and bone formation (Fujita et al., 2004; Zheng et al., 2022). In parallel, MAPK family members—particularly extracellular signal-regulated kinase (ERK) and p38 MAPK—regulate transcription factor activity during the early stages of osteogenic induction, further contributing to lineage commitment and matrix maturation (Hu et al., 2003; Li et al., 2017).

Additionally, oxidative stress and cellular damage frequently arise within the osteogenic microenvironment, posing challenges to osteoblast survival. The mitochondrial apoptosis pathway, particularly the B-cell lymphoma 2 (Bcl-2)/Bcl-2-associated X protein (Bax) ratio regulatory axis, is a key determinant of osteoblast longevity. Upregulation of the anti-apoptotic protein Bcl-2 and downregulation of the pro-apoptotic protein Bax contribute to mitochondrial membrane stability, caspase inhibition, and prolonged osteoblast lifespan, ultimately facilitating bone formation (Jilka et al., 2014).

Natural products such as *Lycium barbarum* polysaccharides (LBPs) extracted from the fruits of *L. barbarum* L. (Solanaceae) have shown promise in supporting osteoblast survival and matrix formation. Recent studies report that LBP enhances both proliferation and osteogenic differentiation of MC3T3-E1 pre-osteoblasts, resulting in improved bone-forming capacity (Li Z-X. et al., 2024). In certain cellular models, LBP appears to mitigate oxidative stress-induced apoptosis by upregulating Bcl-2 and suppressing Bax expression (Xie et al., 2021). However, most mechanistic evidence to date has been derived from non-osteogenic or cancer-related cell types. Direct validation of Bcl-2/Bax regulation by LBP in osteogenic cells—such as BMSCs or MC3T3-E1 cells—remains limited. Addressing this knowledge gap will be essential for substantiating the anti-apoptotic mechanisms of LBP in the context of bone regeneration.

### 3.3 Suppression of osteoclastogenesis via RANKL-RANK-MAPK-NFATc1 signaling

Osteoclast differentiation and activity are essential components of bone resorption during skeletal remodeling. The RANKL–receptor activator of nuclear factor κB (RANK)–osteoprotegerin (OPG) signaling axis serves as the primary regulatory pathway controlling osteoclastogenesis. Upon binding to its receptor RANK on pre-osteoclasts, RANKL activates several downstream cascades, including MAPK family members—such as ERK, c-Jun N-terminal kinase (JNK), and p38—which subsequently induce the expression of nuclear factor of activated T cells c1 (NFATc1), the master transcription factor for osteoclast-specific gene expression (Huang et al., 2006). Inhibition of this signaling pathway effectively suppresses osteoclast formation and bone resorptive activity, offering therapeutic promise in high bone turnover conditions such as osteoporosis.

Multiple natural products have demonstrated the ability to interfere with this signaling axis and exert anti-resorptive effects. For instance, resveratrol, a stilbene polyphenol isolated from the skins of *Vitis vinifera* L. (Vitaceae) and the roots of *Polygonum cuspidatum* Siebold and Zucc. (Polygonaceae), and its hydroxylated analog oxyresveratrol, obtained from the twigs of *Morus alba* L. (Moraceae), have been shown to inhibit the activation of MAPK pathways—particularly p38, JNK, and ERK—during RANKL-induced osteoclast differentiation (Lee et al., 2024). This inhibition leads to the downregulation of key osteoclastogenic genes, including *NFATc1* and *Cathepsin K*, and improves BMD and trabecular architecture in OVX rat models.

Epigallocatechin-3-gallate (EGCG), a major polyphenol in green tea, has been reported to promote osteogenesis by upregulating *Runx2* and *Osterix* while concurrently suppressing osteoclastogenesis via inhibition of the RANKL/NF-κB signaling axis (Lin et al., 2018). This dual action contributes to a favorable microenvironment for bone formation.

In addition, triterpenoids such as oleanolic acid, a pentacyclic triterpenoid isolated from the fruits of *Olea europaea* L. (Oleaceae), have been shown to modulate the RANKL/MAPK signaling pathway, thereby inhibiting osteoclast differentiation and attenuating bone loss (Xie et al., 2019). Similarly, ursolic acid, a structurally related triterpenoid obtained from the peels of *Malus domestica* Borkh. (Rosaceae), suppresses osteoclast formation by blocking NF-κB signaling and downregulating its downstream effector *NFATc1* (Jiang et al., 2015).

Collectively, these findings suggest that natural products can suppress osteoclastogenesis by targeting the RANKL–RANK–MAPK–NFATc1 axis, providing mechanistic insight and pharmacological rationale for their application in the prevention and treatment of bone-resorptive diseases such as osteoporosis.

### 3.4 Coupled regulation of bone remodeling via OPG/RANKL and chemokine signaling

Bone remodeling is a tightly coordinated process governed by the dynamic crosstalk between osteoblasts and osteoclasts. Among the key regulatory pathways, the OPG/RANKL axis and chemokine-mediated cell–cell communication play pivotal roles in maintaining skeletal homeostasis.

The binding of RANKL to its receptor RANK on pre-osteoclasts activates the TNF receptor-associated factor 6 (TRAF6)/NF-κB/NFATc1 signaling cascade, thereby promoting osteoclastogenesis and bone resorption. Conversely, upregulation of OPG—an endogenous decoy receptor—competitively inhibits RANKL–RANK interaction and effectively suppresses osteoclast differentiation and function (Takayanagi et al., 2002).

In parallel, pro-inflammatory conditions at bone remodeling sites often induce the expression of chemokines such as C–C motif ligand 2 (CCL2) and C–X–C motif chemokine ligand 12 (CXCL12). These chemokines promote the migration and local accumulation of osteoclast precursors, further exacerbating bone resorption and disrupting the osteoblast–osteoclast balance (Brylka and Schinke, 2019; Siddiqui and Partridge, 2017). Therefore, targeting RANKL signaling alone may be insufficient in pathological bone conditions; an ideal therapeutic approach should also modulate chemokine-mediated recruitment of osteoclast precursors to restore bone remodeling equilibrium.

Several glycosides derived from TCM have demonstrated dual regulatory effects on bone remodeling by simultaneously enhancing osteogenesis and inhibiting osteoclastogenesis through modulation of these signaling pathways. For instance, diosgenin, a steroidal sapogenin extracted from the tubers of *Dioscorea oppositifolia* L. (Dioscoreaceae), has been shown to upregulate *OPG* and downregulate *RANKL* expression in OXYS rat models, thereby reducing osteoclast activity and improving trabecular bone structure (Tikhonova et al., 2015). Similarly, ophiopogonin D, a steroidal saponin isolated from the tubers of *Ophiopogon japonicus* (L. f.) Ker Gawl. (Asparagaceae), upregulates *OPG* and suppresses *RANKL* in OVX rats, contributing to decreased osteoclast-mediated bone resorption and improved bone microarchitecture (Huang et al., 2015). Cycloastragenol, a triterpenoid aglycone derived from the roots of *Astragalus membranaceus* (Fisch.) Bunge (Fabaceae), has also been reported to inhibit osteoclast formation and activity by suppressing RANKL-induced NF-κB/NFATc1 signaling *in vitro* and *in vivo* (Yu et al., 2020).

**FIGURE 1:**
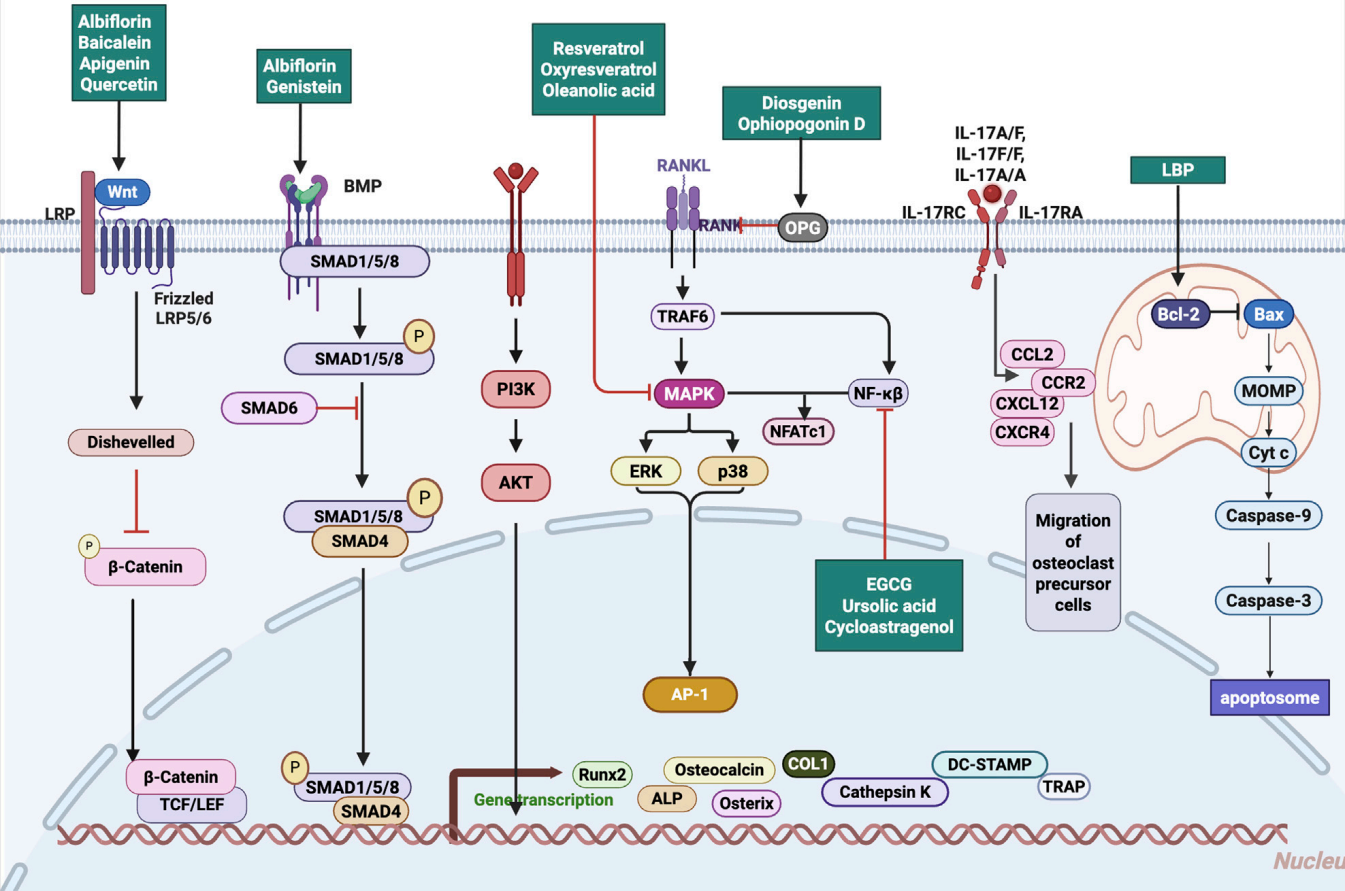
Mechanistic pathways targeted by natural products in regulating bone remodeling. Natural Products target Wnt/β-catenin, BMP/SMAD, PI3K/Akt, and RANKL–NF-κB signaling pathways, modulating osteogenic activation, osteoclastogenesis inhibition, and inflammation suppression. Abbreviations: Wnt, wingless-related integration site; LRP, low-density lipoprotein receptor-related protein; BMP, bone morphogenetic protein; SMAD, mothers against decapentaplegic homolog; PI3K, phosphoinositide 3-kinase; Akt, protein kinase B; MAPK, mitogen-activated protein kinase; ERK, extracellular signal-regulated kinase; AP-1, activator protein 1; NF-κB, nuclear factor kappa-light-chain-enhancer of activated B cells; TRAF6, TNF receptor-associated factor 6; RANK, receptor activator of nuclear factor κB; RANKL, receptor activator of nuclear factor κB ligand; OPG, osteoprotegerin; NFATc1, nuclear factor of activated T cells 1; IL, interleukin; IL-17RC, interleukin-17 receptor C; IL-17RA, interleukin-17 receptor A; CCL2, C-C motif chemokine ligand 2; CCR2, C-C motif chemokine receptor 2; CXCL12, C-X-C motif chemokine ligand 12; CXCR4, C-X-C motif chemokine receptor 4; LBP, lipopolysaccharide-binding protein; Bcl-2, B-cell lymphoma 2; Bax, Bcl-2-associated X protein; MOMP, mitochondrial outer membrane permeabilization; Cyt c, cytochrome c; Caspase-9, cysteine-aspartic acid protease 9; Caspase-3, cysteine-aspartic acid protease 3; ALP, alkaline phosphatase; COL1, collagen type I; Runx2, runt-related transcription factor 2; Osterix, transcription factor Sp7; TRAP, tartrate-resistant acid phosphatase; DC-STAMP, dendritic cell-specific transmembrane protein; TCF/LEF, T cell factor/lymphoid enhancer factor.

While chemokines play critical roles in regulating osteoclast precursor trafficking and mediating osteoblast–osteoclast interactions, direct evidence supporting the modulation of chemotactic signaling by glycosides remains limited. Future studies are warranted to elucidate whether these products influence bone remodeling through interference with chemokine pathways, which could offer additional therapeutic leverage in the treatment of bone resorptive disorders. Natural products modulate osteogenic and osteoclastic signaling pathways, including Wnt/β-catenin, BMP/SMAD, PI3K/Akt, and NF-κB axes, thereby orchestrating bone remodeling and immune balance (Figure 1).

## 4 Bone microenvironmental regulation by natural products

### 4.1 Angiogenesis coupling: VEGF/Notch/eNOS pathways and osteo-vascular crosstalk

In recent years, the mechanistic coupling between osteogenesis and angiogenesis has emerged as a central theme in bone regeneration research. Osteogenesis–angiogenesis coupling refers to the tightly regulated interaction between bone-forming osteoblasts and angiogenic endothelial cells, which ensures synchronized tissue remodeling and vascularization during bone repair. This bidirectional communication is essential for maintaining homeostasis within the bone microenvironment and supporting functional skeletal regeneration.

A growing body of evidence suggests that natural bioactive products derived from TCM can modulate this osteo–vascular crosstalk by targeting key signaling pathways, including Vascular endothelial growth factor (VEGF), Notch, PI3K/Akt, and endothelial nitric oxide synthase (eNOS). These products exert multi-target pro-regenerative effects by influencing both osteogenic and angiogenic processes, making them attractive candidates for treating metabolic bone diseases such as osteoporosis.

Within the Wnt/β-catenin–VEGF axis, oridonin (ORI), an ent-kaurane-type diterpenoid isolated from the aerial parts of *Rabdosia rubescens* (Thunb.) Hara (Lamiaceae), has been shown to activate Wnt3a/β-catenin signaling and upregulate *VEGF* expression, thereby promoting Type H vessel formation and enhancing bone density and structural regeneration (Yu et al., 2023, PMID: 36924567). Similarly, Panax notoginseng saponins (PNSs), a mixture of dammarane-type triterpenoid saponins extracted from the roots of *Panax notoginseng* (Burk.) F.H. Chen (Araliaceae), upregulate *VEGF*, *Angiopoietin-1 (Ang-1)*, and *Vascular endothelial growth factor receptor 2 (VEGFR2)* by activating the PI3K/Akt/mechanistic target of rapamycin (mTOR) pathway, promoting vascular regeneration and callus formation at fracture sites (Jiang et al., 2024).

Astragaloside IV (AS-IV), a triterpenoid saponin isolated from the roots of *Astragalus membranaceu*s (Fisch.) Bunge (Fabaceae), and polydatin (POL), a stilbene glucoside derived from the roots of *P. cuspidatum* Siebold and Zucc. (Polygonaceae), activate the PI3K/Akt/GSK-3β signaling cascade, leading to upregulation of *Runx2* and *Hypoxia-inducible factor-1 alpha (HIF-1α)*, which synergistically enhance osteogenesis and angiogenesis (Wang et al., 2021; Zhou et al., 2025). The MAPK/ERK1/2 pathway also plays a critical role in coupling regulation: icariin (ICA), a prenylated flavonol glycoside isolated from the aerial parts of *Epimedium brevicornum* Maxim. (Berberidaceae), and its metabolite icariside II have been shown to activate this pathway, upregulating *Runx2*, *osteocalcin*, and *VEGF*, thereby promoting both osteogenic differentiation of BMSCs and endothelial migration and tube formation (S et al., 2024). Shikonin, a naphthoquinone derivative isolated from the roots of *Lithospermum erythrorhizon* Siebold and Zucc. (Boraginaceae), has also been increasingly recognized for its modulatory effects within this pathway (Hu et al., 2025).

In addition to direct signaling modulation, certain products act via paracrine mechanisms. Albiflorin, a monoterpene glycoside isolated from the roots of *P. lactiflora* Pall. (Paeoniaceae), and echinacoside, a phenylethanoid glycoside extracted from the stems of *Cistanche tubulosa* (Schenk) Wight (Orobanchaceae), have been shown to promote osteogenesis–angiogenesis coupling by enhancing paracrine signaling in BMSCs, which indirectly induces Type H vessel formation (Sun et al., 2025; Yi et al., 2024).

**FIGURE 2:**
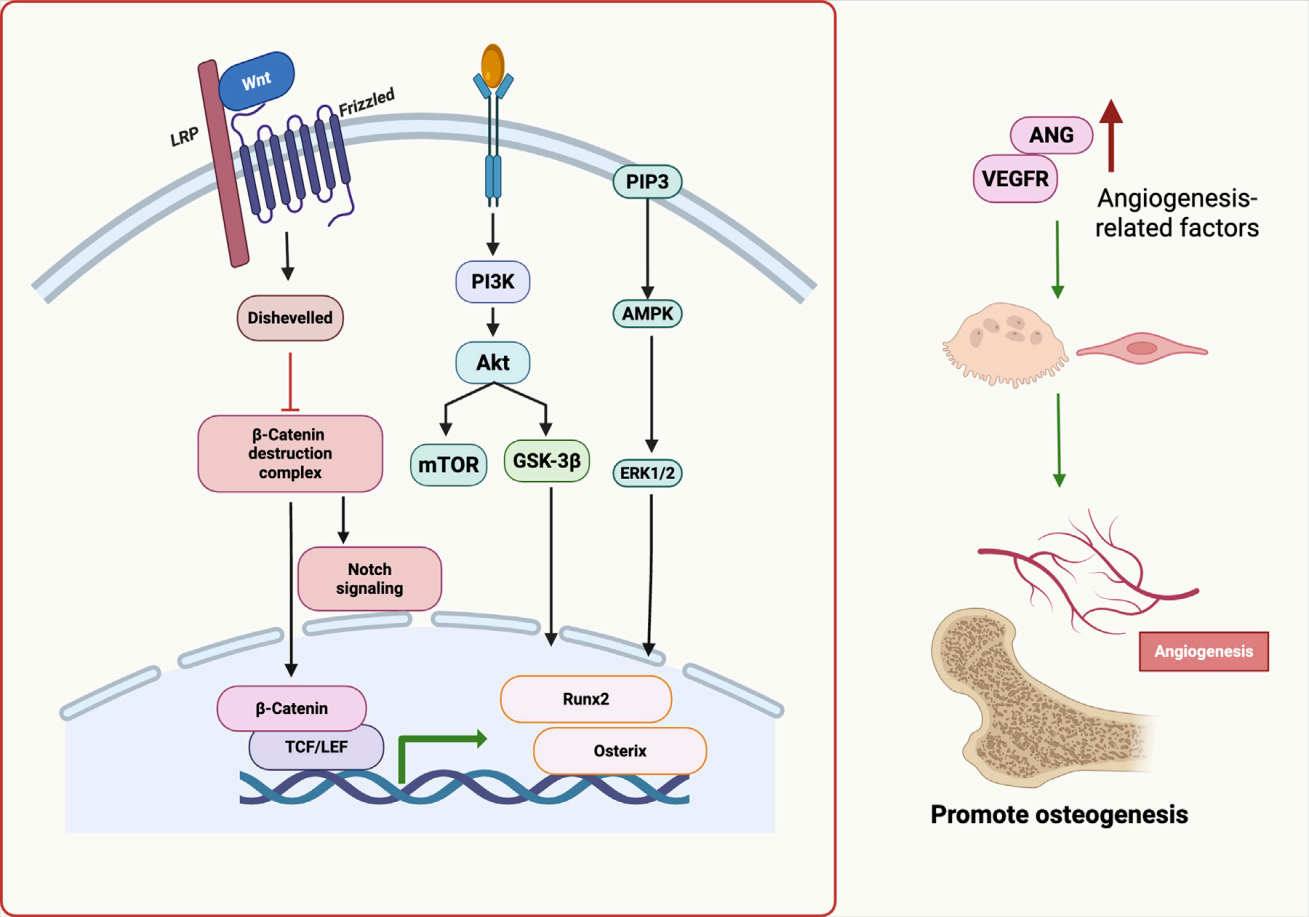
Molecular pathways underlying angiogenesis–osteogenesis coupling regulated by natural products. Natural Products activate Wnt/β-catenin, PI3K/Akt, VEGF, and Notch pathways, promoting osteogenic transcription factors (e.g., Runx2, Osterix) and angiogenic mediators. These synergistic signals enhance osteo-vascular coupling and support bone regeneration. Abbreviations: Wnt, wingless-related integration site; LRP, low-density lipoprotein receptor-related protein; PI3K, phosphoinositide 3-kinase; Akt, protein kinase B; PIP3, phosphatidylinositol (3,4,5)-trisphosphate; AMPK, AMP-activated protein kinase; mTOR, mechanistic target of rapamycin; GSK-3β, glycogen synthase kinase-3 beta; ERK1/2, extracellular signal-regulated kinase 1/2; TCF/LEF, T cell factor/lymphoid enhancer factor; Runx2, runt-related transcription factor 2; Osterix (Osx), transcription factor Sp7; ANG, angiogenin; VEGFR, vascular endothelial growth factor receptor.

Collectively, extensive *in vitro* and *in vivo* evidence demonstrates that natural products modulate osteogenesis–angiogenesis coupling via multiple signaling pathways and cell types. A detailed summary of representative products, their molecular targets, and associated biological effects is provided in Table 1, illustrating the systems-level advantages of TCM in orchestrating the bone regenerative microenvironment. These findings suggest that natural productss enhance osteo-angiogenic coupling by activating Wnt/β-catenin, PI3K/Akt/mTOR, and VEGF-related pathways, thereby promoting bone regeneration (Figure 2).

**TABLE 1:** Posological regimens and experimental models of representative natural metabolites used in osteogenesis–angiogenesis studies.

Drug class	Compound	Botanical source	Type of model	Extract type/Solvent	Working concentration	Signaling pathway/Target	Mechanism	Research stage	References
Flavonoids	Icariin	a prenylated flavonol glycoside isolated from the aerial parts of *Epimedium brevicornum* Maxim. (Berberidaceae)	*In vivo*: Bone defect model in T1DM rats *In vitro*:rat BMSCs	Purified monomer	ICA: 100 mg/kg/day by gavage for 4 weeks (*in vivo*) 1/10/100 μM for 1–7 days (*in vitro*)	Enhances H-type angiogenesis	Osteogenesis-angiogenesis	*In vitro* and *In vivo*	Zheng et al. (2024b)
	Icariin and its metabolite Icariside II	a prenylated flavonol glycoside isolated from the aerial parts of *Epimedium brevicornum* Maxim. (Berberidaceae)	rat BMSCs	Purified monomer	ICA: 10^–5^ mol/L; ICSII: 10^–5^, 10^–7^, 10^–8^ mol/L for 9 days (*in vitro*)	MAPK/ERK1/2	Osteogenesis-angiogenesis	*In vitro*	Yao et al. (2025)
	Engeletin	a flavonoid compound isolated from the aerial parts of *Epimedium sagittatum* (Siebold and Zucc.) Maxim. (Berberidaceae)	Erastin-induced BMSCs ferroptosis model	Purified monomer	Engeletin: 20 μM and 40 μM for 12–24 h (*in vitro*)	Nrf2/Keap1, ↑GPX4, ↓ROS, ↓LPO	Osteogenesis-angiogenesis	*In vitro*	Huang et al. (2023)
	Vitexin	a flavone C-glycoside isolated from the leaves of *Vitex negundo* L. (Lamiaceae) or the fruits of *Crataegus pinnatifida* Bunge (Rosaceae)	*In vivo*: OVX rat model *In vitro*: EAhy926 endothelial cells under hypoxic conditions	Purified monomer	Vitexin: 10 mg/kg/day by gavage for 3 months (*in vivo*) 0–256 μmol/L (*in vitro*)	VDR/eNOS	Osteogenesis-angiogenesis	*In vitro* and *In vivo*	Liu et al. (2023)
	Daidzein	an isoflavone compound isolated from the seeds of *Glycine* max (L.) Merr. (Fabaceae)	OVX-induced osteoporosis mouse model	Purified monomer	Daidzein: 25 mg/kg/day, intragastric administration, 5 days/week for 8 weeks (*in vivo*)	EGFR/AKT/PI3K	Osteogenesis-angiogenesis	*In vivo*	Jia et al. (2023)
	total flavonoids of *Rhizoma Drynariae*	Total flavonoids extracted from the rhizomes of *Drynaria fortunei* (Kunze ex Mett.) J. Sm. (Polypodiaceae)	*In vivo*: Tibial distraction osteogenesis model in adult male Sprague-Dawley rats *In vitro*: EPCs and BMSCs under stress conditions	Methanol extract (for LC-MS preparation and analysis)	TFRD: 75 mg/kg/day, oral gavage, once daily from Day 1 post-surgery (*in vivo*) 100 μg/mL (*in vitro*)	PDGF-BB,VEGF, RUNX2,OSX	Osteogenesis-angiogenesis	*In vitro* and *In vivo*	Shen et al. (2022)
Saponins/Glycosides	Astragaloside IV	a triterpenoid saponin isolated from the roots of *Astragalus membranaceus* (Fisch.) Bunge (Fabaceae)	*In vivo*: Tibial distraction osteogenesis model *In vitro*: BMSCs	Purified monomer	AS-IV: 20 mg/kg/day by intragastric gavage during consolidation phase (*in vivo*) AS-IV: 0–80 μM,optimal: 40 μM (*in vitro*)	AKT/GSK-3β/β-catenin	Osteogenesis-angiogenesis-immune coupling	*In vitro* and *In vivo*	Wang et al. (2021)
			*In vivo*: Steroid-induced Avascular Necrosis of the Femoral Head rat model (male Sprague–Dawley rats, 8 weeks old) *In vitro*: BMSCs and HUVECs	Purified monomer	AS-IV:20 mg/kg/day by gavage (*in vivo*) 5, 20, 50, 100 μM (*in vitro*)	PI3K/Akt	Promotes osteogenesis, angiogenesis, anti-apoptosis, and anti-oxidation	*In vitro* and *In vivo*	Shan et al. (2023)
	Echinacoside	a phenylethanoid glycoside isolated from the stems of *Cistanche tubulosa* (Schenk) R. Wight (Orobanchaceae)	*In vivo*: Rat fracture model *In vitro*: MC3T3-E1 (osteoblast precursor), HUVECs, RAW264.7 (osteoclast precursor)	Purified monomer	Echinacoside: 2–256 μg/mL (proliferation); 64, 128 μg/mL (morphology, differentiation) (*in vitro*); *in vivo* dose not stated	↑(VEGF、RUNX2、OCN); ↓(MMP-9、CTSK); ↓(TNF-α、IL-1β、IL-6)	Osteogenesis-angiogenesis-immune coupling	*In vitro* and *In vivo*	Yi et al. (2024)
	Ginsenoside Rg1	a triterpenoid saponin isolated from the roots of *Panax ginseng* C.A. Mey. (Araliaceae)	*In vivo*: Goto-Kakizaki diabetic rat model *In vitro*: Osteoprogenitors and HUVECs under high-glucose (32.8 mM) conditions	Purified monomer	GR1:10 mg/kg/day by oral gavage for 12 weeks (*in vivo*) 164.8 μM (*in vitro*)	Notch/Noggin	Osteogenesis-angiogenesis	*In vitro* and *In vivo*	Chen W et al. (2022)
	compound K	Intestinal metabolite derived from the deglycosylation of *Panax ginseng* C.A. Mey. (Araliaceae) saponins in the gut microbiota	*In vivo*: Open femoral fracture model with intramedullary fixation in SD ratsIn vitro: BMSCs and HUVECs	Purified monomer	CK: 500 μM, local injection every other day for 4 weeks (*in vivo*); 0–40 μM, optimal at 30 μM (*in vitro*)	Wnt/β-catenin	Osteogenesis-angiogenesis	*In vitro* and *In vivo*	Ding et al. (2022)
	Aucubin	an iridoid glycoside isolated from the leaves of *Aucuba japonica* Thunb. (Garryaceae)	*In vivo*: RANKL-induced osteoporotic medaka and vascular zebrafish (Tg (fli1a-EGFP)) models *In vitro*: HUVECs ± SU5416-induced endothelial injury	Purified monomer	Aucubin: 25–50 μM by immersion for 5 days (*in vivo*); 3.13–50 μM for 48 h (*in vitro*)	VEGF-VEGFR、Akt/mTOR	Osteogenesis-angiogenesis	*In vitro* and *In vivo*	He et al. (2023)
			*In vivo*: OVX-induced osteoporosis model in BALB/c mice *In vitro*: RAW264.7 (RANKL-induced osteoclastogenesis) and MMECs	Purified monomer	Aucubin: 5 mg/kg i.p. every 2 days for 4 weeks (*in vivo*); 0, 1, or 5 μM (*in vitro*)	MAPK/NF-κB inhibition, ↑PDGF-BB	Osteogenesis-angiogenesis	*In vitro* and *In vivo*	Li et al. (2022)
	Panax notoginseng saponins	a mixture of dammarane-type triterpenoid saponins extracted from the roots of Panax notoginseng (Burk.) F.H. Chen (Araliaceae)	*In vivo*: OVX + tibial fracture model (SD rats)	Purified monomer	PNS: 100 or 200 mg/kg/day, intraperitoneal injection for 7, 14, or 21 days (*in vivo*)	PI3K/AKT/mTOR	Osteogenesis-angiogenesis	*In vivo*	Jiang et al. (2024)
	Albiflorin	a monoterpene glycoside isolated from the roots of *Paeonia lactiflora* Pall. (Paeoniaceae)	*In vivo*: OVX + drill-hole defect model (SD rats) *In vitro*: BMSCs	Purified monomer	ALB: 10 mg/kg/day, intraperitoneal injection for 4 weeks (*in vivo*); 0.01–10 μM (proliferation and osteogenic differentiation) (*in vitro*)	Enhances H-type angiogenesis	Osteogenesis-angiogenesis	*In vitro* and *In vivo*	Sun et al. (2025)
	Asperosaponin VI	a triterpenoid saponin isolated from the roots of *Dipsacus asper* Wall. ex Henry (Caprifoliaceae)	BMSCs (osteogenic differentiation model), BMMs (RANKL + M-CSF-induced osteoclastogenesis)	Purified monomer	ASP VI: 10^–7^ to 10^–3^ M (*in vitro*); optimal range <10^–4^ M	SMADs, TGF-β1, VEGFA, OPG/RANKL	Osteogenesis-angiogenesis-anti-osteoclast	*In vitro*	Chen F et al. (2022)
Phenolic Acids/Polyphenols	Punicalagin	an ellagitannin polyphenol extracted from the peels of *Punica granatum* L. (Lythraceae)	*In vivo*: OVX + tibial fracture model (SD rats)	Purified monomer	PNS: 100 or 200 mg/kg/day, intraperitoneal injection (*in vivo*)	Nrf2/HO-1	Osteogenesis-angiogenesis-immune coupling	*In vivo*	Huang et al. (2023)
Phenolic Acids/Polyphenols	Polydatin	a stilbene glucoside isolated from the roots of *Polygonum cuspidatum* Siebold and Zucc. (Polygonaceae)	*In vivo*: OVX + drill-hole defect model (SD rats) *In vitro*: BMSCs	Purified monomer	POL:40 mg/kg/day, oral gavage for 4 weeks (*in vivo*); 0.1–100 μM (proliferation); 1, 10 μM (osteogenic differentiation) (*in vitro*)	PI3K/AKT/GSK-3β/β-catenin	Osteogenesis-angiogenesis	*In vitro* and *In vivo*	Zhou et al. (2025)
	Resveratrol	a stilbene polyphenol isolated from the skins of *Vitis vinifera* L. (Vitaceae)	*In vitro*:RAW 264.7 macrophages (H_2_O_2_-induced senescence model); EA.hy926 endothelial cells (H_2_O_2_-induced senescence model)	Purified monomer	0.1–1 μM (senescent macrophages) (*in vitro*)	Autophagy of aged macrophages	Osteogenesis-angiogenesis-immune coupling	*In vitro*	Hang et al. (2022)
	Picein	a phenolic glycoside isolated from the bark of *Picea abies* (L.) H. Karst. (Pinaceae)	*In vivo*: OVX + drill-hole defect model (SD rats) *In vitro*: BMSCs (erastin-induced oxidative stress model); HUVECs	Purified monomer	Picein: 40, 80 mg/kg/day, local injection for 4 weeks (*in vivo*); 0–320 μM (viability); 40, 80 μM (oxidative protection) (*in vitro*)	Nrf2/HO-1/GPX4	Osteogenesis-angiogenesis-immune coupling	*In vitro* and *In vivo*	Huang et al. (2024)
	Crocin	a carotenoid glycoside extracted from the stigmas of *Crocus sativus* L. (Iridaceae)	*In vitro*: BMSCs (osteogenesis and oxidative stress); HUVECs (angiogenesis)	Purified monomer	Crocin: 10–100 μM (CCK-8, 48 h); 10 μM (EdU); 10 μM (ROS assay); 10–100 μM (general antioxidant and osteogenic/angiogenic function)	Nrf2/GPX4	Osteogenesis-angiogenesis	*In vitro*	Hong et al. (2025)
	Curcumin	a diarylheptanoid polyphenol derived from the rhizomes of *Curcuma longa* L. (Zingiberaceae)	*In vivo*: STZ-induced diabetic osteoporosis (C57BL/6 mice) *In vitro*: BMSCs under normal/high glucose conditions	Purified monomer	Curcumin: 100 mg/kg/day, oral gavage for 8 weeks (*in vivo*); 0.1–10 μM (proliferation); 1 μM (osteogenic differentiation) (*in vitro*)	NF-κB inhibition	Osteogenesis-angiogenesis	*In vitro* and *In vivo*	Fan et al. (2022)
	Shikonin	a naphthoquinone derivative isolated from the roots of *Lithospermum erythrorhizon* Siebold and Zucc. (Boraginaceae)	*In vivo*: Aged mice (18-month-old C57BL/6) *In vitro*: BMSCs and MC3T3-E1 cells	Purified monomer	Shikonin: 3 mg/kg, i.v., twice weekly for 2 months (*in vivo*); 0.1–0.4 μM (*in vitro*)	MAPK(p38、ERK、JNK)	Osteogenesis-angiogenesis	*In vitro* and *In vivo*	Hu et al. (2025)
Polysaccharides	Astragalus polysaccharide	a natural polysaccharide extracted from the roots of *Astragalus membranaceus* (Fisch.) Bunge (Fabaceae)	Co-culture of outgrowth endothelial cells (OECs, from peripheral blood) and primary human osteoblasts (POBs, from surgical bone fragments)	Purified monomer	APS: 200, 400, 800 μg/mL for 24 h (viability/proliferation); 400 μg/mL optimal (angiogenesis and ossification in co-culture)	TLR4/MyD88	Osteogenesis-angiogenesis	*In vitro*	Qiu et al. (2022)
Coumarins	Osthole	a coumarin derivative extracted from the fruits of *Cnidium monnieri* (L.) Cusson (Apiaceae)	*In vivo*: OVX + tibial fracture model (SD rats) *In vitro*: BMSCs	Purified monomer	Osthole: 10 mg/kg/day, oral gavage for 6 weeks (*in vivo*); 1, 10, 100 μM (*in vitro*)	Wnt/β-catenin	Osteogenesis-angiogenesis	*In vitro* and *In vivo*	Zheng et al. (2024a)
Terpenoids/Others	Oridonin	an ent-kaurane-type diterpenoid isolated from the aerial parts of *Rabdosia rubescens* (Hemsl.) H. Hara (Lamiaceae)	*In vivo*: OVX model (C57BL/6 mice)	Purified monomer	Oridonin (ORI): 10, 20, 40 mg/kg/day, oral gavage for 6 weeks (*in vivo*)	Wnt3a/β-catenin	Osteogenesis-angiogenesis-immune coupling	*In vivo*	Yu et al. (2023)
	Sarsasapogenin	a steroidal sapogenin isolated from the rhizomes of *Anemarrhena asphodeloides* Bunge (Asparagaceae)	*In vivo*: OVX model + GPX4-KO mice (C57BL/6) *In vitro*: BMSCs + HUVECs (ferroptosis induced by iron dextran or erastin)	Purified monomer	SAR: 5, 10 mg/kg/day, oral gavage for 12 weeks (*in vivo*); 0.1, 1 μM (iron dextran- or erastin-induced ferroptosis models in BMSCs; BMSCs-CM for HUVECs assays) (*in vitro*)	GPX4/SLIT3/ROBO1	Osteogenesis-angiogenesis	*In vitro* and *In vivo*	(F et al., 2025)
	Catalpol	an iridoid glycoside isolated from the roots of *Rehmannia glutinosa* (Gaertn.) DC. (Orobanchaceae)	*In vitro*: BMSCs + RAW264.7 macrophages	Purified monomer	Catalpol: 25, 50, 100 μg/mL (*in vitro* proliferation, osteogenic induction, polarization, and anti-inflammation)	M2 polarization, paracrine effects	Osteogenesis-angiogenesis-immune coupling	*In vitro*	Zhang et al. (2022)
Flavonoids	Naringenin	a flavanone aglycone extracted from the peels of *Citrus aurantium* L. (Rutaceae)	*In vivo*: Hindlimb unloading-induced simulated microgravity (SMG) model (C57BL/6J mice) *In vitro*: OBs under 2D-RWVS SMG	Purified monomer	NAR: 60 or 100 mg/kg/day, oral gavage for 4 weeks (*in vivo*); concentration not specified (*in vitro*)	Nrf2/HO-1, Wnt/β-catenin, PI3K/Akt	Osteogenesis-antioxidation	*In vitro* and *In vivo*	Cao et al. (2024)
			*In vitro*: MC3T3-E1 cells under H_2_O_2_-induced oxidative stress	Purified monomer	Nar: 0.1 μM (optimal concentration under 0–1000 μM gradient) (*in vitro*)	PI3K/Akt, Wnt/β-catenin	Osteogenesis-antioxidation	*In vitro*	Wang H et al. (2024)
	Neobavaisoflavone	an isoflavone compound isolated from the seeds of *Psoralea corylifolia* L. (Fabaceae)	*In vitro*: MC-3T3-E1 (Dex-induced oxidative injury model)	Purified monomer	NBIF: 16 μM (protective concentration, pre-treatment for 24 h, followed by Dex 20 μM exposure) (*in vitro*)	lncRNA CRNDE, Nrf2/HO-1	Osteogenesis-antioxidation	*In vitro*	Zhu P et al. (2021)
Saponins/Glycosides	Picein	a phenolic glycoside isolated from the bark of *Picea abies* (L.) H. Karst. (Pinaceae)	*In vivo*: OVX-induced osteoporotic bone defect model (SD rats) *In vitro*: BMSCs + RAW264.7 macrophages + HUVECs	Purified monomer	Picein: 40–80 μM (protection against oxidative stress, osteogenesis, M2 polarization, angiogenesis) (*in vitro*)	Nrf2/HO-1/GPX4	Osteogenesis-antioxidation	*In vitro* and *In vivo*	Huang et al. (2024)
	Curculigoside	a phenolic glycoside isolated from the rhizomes of *Curculigo orchioides* Gaertn. (Hypoxidaceae)	*In vitro*: RAW264.7 (RANKL + H_2_O_2_-induced osteoclast model)	Purified monomer	Cur: 1, 5, 10 μM (*in* *vitro*, 72 h, ±H_2_O_2_ 20 μM)	Nrf2, NF-κB	Anti-osteoclastogenesis	*In vitro*	Liu et al. (2021)
	Geniposide	an iridoid glycoside extracted from the fruits of *Gardenia jasminoides* J. Ellis (Rubiaceae)	*In vitro*: MC3T3-E1 cells under CdCl_2_-induced oxidative injury	Purified monomer	Geniposide: 100, 200, 400 μg/mL (*in vitro*)	Nrf2/HO-1/NQO1	Osteoblast protection	*In vitro*	He et al. (2019)
	Catalpol	an iridoid glycoside isolated from the roots of *Rehmannia glutinosa* (Gaertn.) DC. (Orobanchaceae)	*In vivo*: Subcutaneous implantation model (SD rats, electrospun PLA/gelatin scaffold) *In vitro*: RAW264.7 + BMSCs	Purified monomer	Catalpol: 25, 50, 100 μg/mL (*in vitro*); incorporated into scaffold, no fixed dosage (*in vivo*)	M1/M2 polarization, paracrine regulation	Osteogenesis-immunoregulation	*In vitro* and *In vivo*	Zhang et al. (2022)
Phenolic Acids/Polyphenols	Curcumin	a diarylheptanoid polyphenol derived from the rhizomes of *Curcuma longa* L. (Zingiberaceae)	*In vitro*: MC3T3-E1 under H_2_O_2_-induced oxidative stress	Purified monomer	Curcumin: 0.25 μM, pretreatment for 24 h (*in vitro*)	GSK3β-Nrf2	Antioxidation, protection against osteoblast dysfunction	*In vitro*	Li B et al. (2020)
			*In vivo*: LPS + Methylprednisolone-induced steroid-associated osteonecrosis model (C57BL/6 mice) *In vitro*: RAW264.7 (M1-polarized) + MLO-Y4 co-culture model	Purified monomer	Curcumin: 100 mg/kg/day, oral gavage for 4 weeks (*in vivo*) 6.25, 12.5, 25 μM (*in vitro*)	JAK1/2-STAT1, M1 macrophage polarization	Anti-inflammation, anti-apoptosis of osteocytes	*In vitro* and *In vivo*	Jin et al. (2020)
	Apocynin	a methoxy-substituted catechol compound isolated from the roots of *Apocynum cannabinum* L. (Apocynaceae)	Apocynin exerts cytoprotective effects on dexamethasone-induced osteoblasts by inhibiting oxidative stress through the Nrf2 signalling pathway	Purified monomer	APO: 100 mg/kg/day, oral gavage for 4 weeks (*in vivo*); 1–100 μM (*in vitro*)	Nrf2	Osteoblast protection	*In vitro* and *In vivo*	Zhang et al. (2023)
Polysaccharides	Grifola frondosa polysaccharide	a natural β-glucan polysaccharide extracted from the fruiting bodies of *Grifola frondosa* (Dicks.) Gray (Polyporaceae)	*In vivo*: OVX model (C57BL/6 mice)	Purified water-soluble polysaccharide via hot water extraction + ethanol precipitation	GFP: 10, 20, 40 mg/kg/day, oral gavage for 6 weeks (*in vivo*)	PINK1/Parkin, IL-6, TNF-α	Anti-inflammatory, antioxidation	*In vivo*	Liu et al. (2023)

Viewed in combination, botanical drugs facilitate the coupling of osteogenesis and angiogenesis via activation of VEGF, PI3K/Akt, Notch, and eNOS signaling. This dual regulatory strategy not only offers promising therapeutic avenues for metabolic bone diseases but also provides a theoretical foundation for the development of vascularized bone constructs in tissue engineering. Future research should focus on elucidating the precise regulatory mechanisms of representative products across these signaling axes, with particular attention to the dynamic interplay between osteogenic and endothelial cell populations. Such efforts will enhance our understanding of the precision, reproducibility, and translational potential of botanical interventions in bone regeneration.

#### 4.1.1 Flavonoids

Natural flavonoids represent one of the most extensively studied classes of bioactive products in TCM. In recent years, they have gained increasing attention for their dual regulatory roles in osteogenesis–angiogenesis coupling, as they promote both osteogenic differentiation and angiogenic activity during bone regeneration.

Numerous studies have shown that flavonoids not only stimulate osteogenic differentiation of BMSCs, but also enhance the functional activity of vascular endothelial cells. This bidirectional modulation strengthens osteo–vascular crosstalk, making these products highly relevant for the repair of bone defects and the treatment of osteoporosis.

ICA has been reported to activate the MAPK/ERK1/2 signaling pathway, leading to upregulation of osteogenic markers such as *ALP*, *Runx2*, and *OCN*, alongside angiogenic factors including *VEGF* and *Cluster of Differentiation 31 (CD31)*. ICA significantly promotes the formation of CD31hiEmcnhi (Type H) vessels and enhances new bone formation, demonstrating therapeutic efficacy in diabetic bone defect models. Its major metabolite, icariside II, exhibits similar effects via the same pathway, supporting the stability and reproducibility of this signaling mechanism (S et al., 2024).

In addition to ICA, several other flavonoids have also demonstrated coordinated regulatory effects on osteogenesis and angiogenesis. Vitexin, a flavone C-glycoside isolated from the leaves of *Vitex negundo* L. (Lamiaceae), enhances endothelial cell migration and tube formation through activation of the vitamin D receptor (VDR)/eNOS signaling pathway. *In vivo*, it mitigates bone loss in OVX models, underscoring its potential in vascular-dependent osteogenesis, particularly for the prevention and treatment of postmenopausal OP (Liu et al., 2023). Daidzein, an isoflavone predominantly found in the seeds of *G.* max (L.) Merr. (Fabaceae), has been shown to promote the proliferation and migration of bone marrow endothelial cells by downregulating Caveolin-1 and activating the Epidermal growth factor receptor (EGFR)/PI3K/Akt signaling cascade. This effect leads to enhanced formation of H-type capillaries in trabecular bone and significantly alleviates OVX-induced bone loss (Jia et al., 2023). Furthermore, in a distraction osteogenesis model, the total flavonoids extracted from the rhizome of *Drynaria fortunei (Kunze ex Mett.)* J.Sm. (Polypodiaceae) (TFRD) have been reported to simultaneously activate endothelial progenitor cells and BMSCs under conditions of mechanical loading. This dual activation is mediated through the Platelet-derived growth factor-BB (PDGF-BB)/VEGF/RUNX2/Osterix (OSX) signaling axis and results in a significant increase in CD31hiEmcnhi Type H vessel formation, ultimately accelerating bone regeneration (Shen et al., 2022).

Despite the promising evidence supporting flavonoids in promoting osteo–angiogenic coupling, their *in vivo* stability, limited bioavailability, and pharmacokinetic variability remain major challenges for clinical translation. Further optimization through structural modification or biomaterial-assisted delivery strategies may help to overcome these limitations.

#### 4.1.2 Glycosides and saponins

Natural glycosides and saponins exhibit dual regulatory activities by simultaneously promoting osteogenesis and angiogenesis, making them promising candidates for the prevention and treatment of bone metabolic disorders. Emerging evidence suggests that these products modulate key signaling pathways that coordinate osteoblastogenesis, angiogenesis, and osteoclastogenesis, thus positioning them as important molecular regulators in osteo–vascular coupling.

AS-IV is one of the most well-characterized products in this category. It has shown consistent osteo-vascular protective effects in both glucocorticoid-induced OP and distraction osteogenesis models (Shan et al., 2023; Wang et al., 2021). Mechanistically, AS-IV promotes CD31hiEmcnhi (Type H) vessel formation by preserving pre-osteoclasts and enhancing PDGF-BB secretion. Concurrently, it facilitates the osteogenic differentiation of BMSCs via multiple signaling pathways, including AKT/GSK-3β/β-catenin, Akt/Runx2, and Akt/HIF-1α/VEGF. Furthermore, AS-IV helps maintain osteo-vascular homeostasis by attenuating oxidative stress and inhibiting apoptosis through activation of the Akt/Nrf2/HO-1 pathway (Shan et al., 2023; Wang et al., 2021).

Echinacoside has also been reported to enhance both angiogenic and osteogenic responses via activation of the VEGF/RUNX2 axis. In addition, it suppresses osteoclastogenesis by downregulating *cathepsin K (CTSK)* and *Matrix metalloproteinase-9 (MMP-9)* expression and attenuating pro-inflammatory cytokine release. In fracture models, echinacoside accelerates bone regeneration through its combined pro-osteogenic, anti-inflammatory, and pro-angiogenic actions (Yi et al., 2024).

Ginsenoside Rg1 (Rg1), a triterpenoid saponin isolated from the roots of *Panax ginseng* C.A. Mey. (Araliaceae), and its metabolite compound K(CK) have demonstrated the ability to enhance osteogenesis and angiogenesis by activating the Notch and Wnt/β-catenin pathways in BMSCs and Human umbilical vein endothelial cells (HUVECs), respectively. These actions promote Type H vessel formation and bone structural repair, supporting a bidirectional regulatory loop between osteoblasts and endothelial cells (Chen F et al., 2022; Ding et al., 2022).

Additionally, aucubin, an iridoid glycoside isolated from the leaves of *Eucommia ulmoides* Oliv. (Eucommiaceae), exhibits indirect osteogenic effects through the coordination of anti-resorptive and pro-angiogenic mechanisms. In OVX rat models, Aucubin suppresses the MAPK/NF-κB pathway and preserves pre-osteoclasts, thereby promoting PDGF-BB–mediated Type H vessel formation and improving bone microarchitecture (He et al., 2023; Li et al., 2022).

PNS activate the PI3K/Akt/mTOR pathway and upregulate multiple angiogenic mediators, including *VEGF*, *Ang-1*, *VEGFR2*, and *Angiopoietin-like protein 2 (ANGPTL2)*. These effects enhance local vascularization and facilitate callus formation at fracture sites. The dual targeting properties of PNS in osteogenesis and angiogenesis have been validated in multiple preclinical models, rendering it one of the most extensively studied products in the context of osteo–vascular coupling (Jiang et al., 2024).

Moreover, albiflorin has been shown to stimulate the paracrine activity of BMSCs, which in turn promotes angiogenesis in HUVECs and facilitates the formation of CD31hiEMCNhi vessels. This mechanism represents a distinct paracrine regulatory strategy, wherein osteogenic cells indirectly modulate endothelial behavior, contrasting with conventional direct pro-angiogenic mechanisms (Sun et al., 2025).

In summary, natural flavone glycosides and saponins promote bone–vascular coupling through the activation of multiple signaling pathways, including AKT/GSK-3β, Wnt/β-catenin, and PI3K/Akt/mTOR. These products exert coordinated effects on osteoblast differentiation, endothelial activation, and osteoclast inhibition, establishing a multifaceted regulatory network. Their mechanisms of action have been supported by a growing body of *in vitro* and *in vivo* evidence, providing a robust scientific rationale for their application in OP therapy and bone defect repair. However, limitations such as poor oral absorption, low bioavailability, and batch-to-batch variability in product purity remain critical challenges for clinical translation and should be carefully addressed in future research.

#### 4.1.3 Phenolics and other representative products

Natural phenolic products and their structurally diverse derivatives have attracted considerable interest in bone metabolism research due to their notable antioxidant, anti-inflammatory, and dual modulatory effects on osteogenesis and angiogenesis. In contrast to flavonoids and glycosides, phenolics often exhibit both cytoprotective and metabolic regulatory properties. These products can enhance osteoblast activity and endothelial function via multiple signaling pathways, thereby contributing to the osteogenesis–angiogenesis coupling process. Additionally, several structurally atypical natural products—including coumarins, iridoid glycosides, polysaccharides, and steroidal saponins—have recently demonstrated synergistic regulatory effects on bone and vascular regeneration, thereby expanding the molecular landscape of osteo–vascular crosstalk.

POL a natural stilbenoid, has been shown to promote osteogenic differentiation in BMSCs and upregulate angiogenesis-related gene expression through activation of the PI3K/Akt/GSK-3β/β-catenin signaling pathway (Zhou et al., 2025). Furthermore, POL enhances the angiogenic potential of HUVECs via BMSC-mediated paracrine mechanisms, which are attenuated upon PI3K inhibition. In osteoporotic animal models, POL increases Type H vessel density and promotes new bone formation.

Curcumin, a diarylheptanoid polyphenol isolated from the rhizomes of *Curcuma longa* L. (Zingiberaceae), primarily acts by inhibiting NF-κB signaling under hyperglycemic conditions, thereby restoring the osteogenic and angiogenic functions of BMSCs. In diabetic OP models, curcumin has been shown to alleviate bone loss while increasing Type H vessel formation (Fan et al., 2022).

Beyond phenolic structures, several non-typical natural products also exert bone–vascular regulatory effects via distinct mechanisms. Astragalus polysaccharide (APS), a natural polysaccharide isolated from the roots of *A. membranaceus* (Fisch.) Bunge (Fabaceae),significantly promotes microvascular development by increasing *VEGF* and *PDGF-BB* expression and stimulating the formation of CD31^+^ endothelial structures in BMSC–HUVEC co-culture systems (Qiu et al., 2022).

Osthole, a coumarin derivative isolated from the fruits of *Cnidium monnieri* (L.) Cusson (Apiaceae), while not acting directly on endothelial cells, activates the Wnt/β-catenin pathway in BMSCs and indirectly promotes Type H vessel formation (Zheng et al., 2024a).

Sarsasapogenin (SAR), a steroidal sapogenin isolated from the rhizomes of *Anemarrhena asphodeloides* Bunge (Asparagaceae), facilitates coordinated osteo-angiogenic regeneration by upregulating GPX4 and activating the GPX4/S Slit guidance ligand 3 (SLIT3)/Roundabout guidance receptor 1 (ROBO1) axis. This signaling promotes *SLIT3* secretion by BMSCs and upregulates *ROBO1* expression in HUVECs, thereby enhancing both osteogenesis and angiogenesis. Notably, this effect remains partially preserved in GPX4-deficient models, highlighting SAR as a rare natural product integrating ferroptosis modulation into the osteo–vascular coupling framework (F et al., 2025).

Current evidence suggests that phenolic and structurally diverse natural products possess promising potential for modulating osteogenesis–angiogenesis coupling. However, they vary considerably in potency, mechanistic scope, innovation, and evidentiary maturity. Among them, agents such as POL, ORI, and APS have demonstrated consistent regenerative effects in preclinical models. Meanwhile, sarsasapogenin introduces ferroptosis as a regulatory axis through GPX4/SLIT3/ROBO1 signaling, while catalpol, an iridoid glycoside isolated from the roots of *Rehmannia glutinosa* (Gaertn.) DC. (Orobanchaceae), has been reported to connect macrophages, osteoblasts, and endothelial cells via an immune polarization–paracrine mechanism—both representing mechanistically novel advances.

Despite these developments, this effect was only demonstrated *in vitro* and may be subject to Pan-assay interference compounds (PAINS)-related artifacts. The overall level of evidence remains limited, with most data derived from *in vitro* and animal models. Pharmacokinetic profiles, safety assessments, and translational studies are still lacking, which hinders clinical advancement.

Considering the evidence as a whole, natural phenolics and structurally atypical products regulate osteogenesis and angiogenesis through diverse signaling cascades and multi-cellular interactions. They represent a mechanistically grounded and multifaceted therapeutic strategy for conditions such as OP and bone defects. Future research should aim to integrate these complex regulatory networks, particularly in the emerging domains of immune modulation, ferroptosis, and metabolic reprogramming. Building a comprehensive bone–vascular–immune regenerative framework and advancing high-quality clinical studies with dual endpoints—targeting both BMD and vascularization—will be essential for translating basic discoveries into precision therapies.

### 4.2 Immune modulation: M1/M2 polarization, treg induction, and NF-κB suppression

Bone regeneration is governed not only by the differentiation potential of mesenchymal stem cells and the functional state of osteoblasts but also by the broader regulatory landscape of the bone microenvironment (Dalle Carbonare et al., 2025). This microenvironment constitutes a highly dynamic and interactive network comprising both cellular and acellular components. Key cellular players include osteoblasts, osteoclasts, BMSCs, macrophages, T lymphocytes, and dendritic cells. Non-cellular elements such as oxygen tension, ROS, cytokines, and the ECM also play vital roles in modulating bone metabolism (Kim et al., 2020).

With the advent of the **“**osteoimmunology” concept, the traditional osteoblast–osteoclast binary model has evolved into a more complex bone–immune–vascular regulatory triad (Guder et al., 2020). Bone tissue is now recognized not only as a structural entity for mineralized matrix production and remodeling but also as an immunologically active organ. Resident immune cells continuously interact with skeletal cells via direct contact and paracrine signaling to regulate bone formation, resorption, and regeneration (Yang and Liu, 2021).

In metabolic bone disorders such as osteoporosis, immune dysregulation is often a central pathogenic factor. A shift toward pro-inflammatory immune phenotypes—such as increased M1-type macrophage infiltration and elevated levels of cytokines like Tumor necrosis factor-alpha (TNF-α) and Interleukin-6 (IL-6)—has been shown to inhibit osteogenesis, stimulate osteoclastogenesis, and ultimately accelerate bone loss (Sun et al., 2021). These cytokines suppress Runx2 differentiation of BMSCs. Likewise, Tregs can suppress excessive immune activation while secreting osteoinductive cytokines and enhancing osteoblast survival.

Furthermore, the NF-κB signaling pathway, a central mediator of inflammation, has been identified as a critical target in immune–bone crosstalk. Its sustained activation under inflammatory conditions inhibits osteoblast differentiation and promotes osteoclastogenesis. As such, NF-κB suppression has become a key therapeutic objective in modulating the immune microenvironment for bone repair.

**FIGURE 3:**
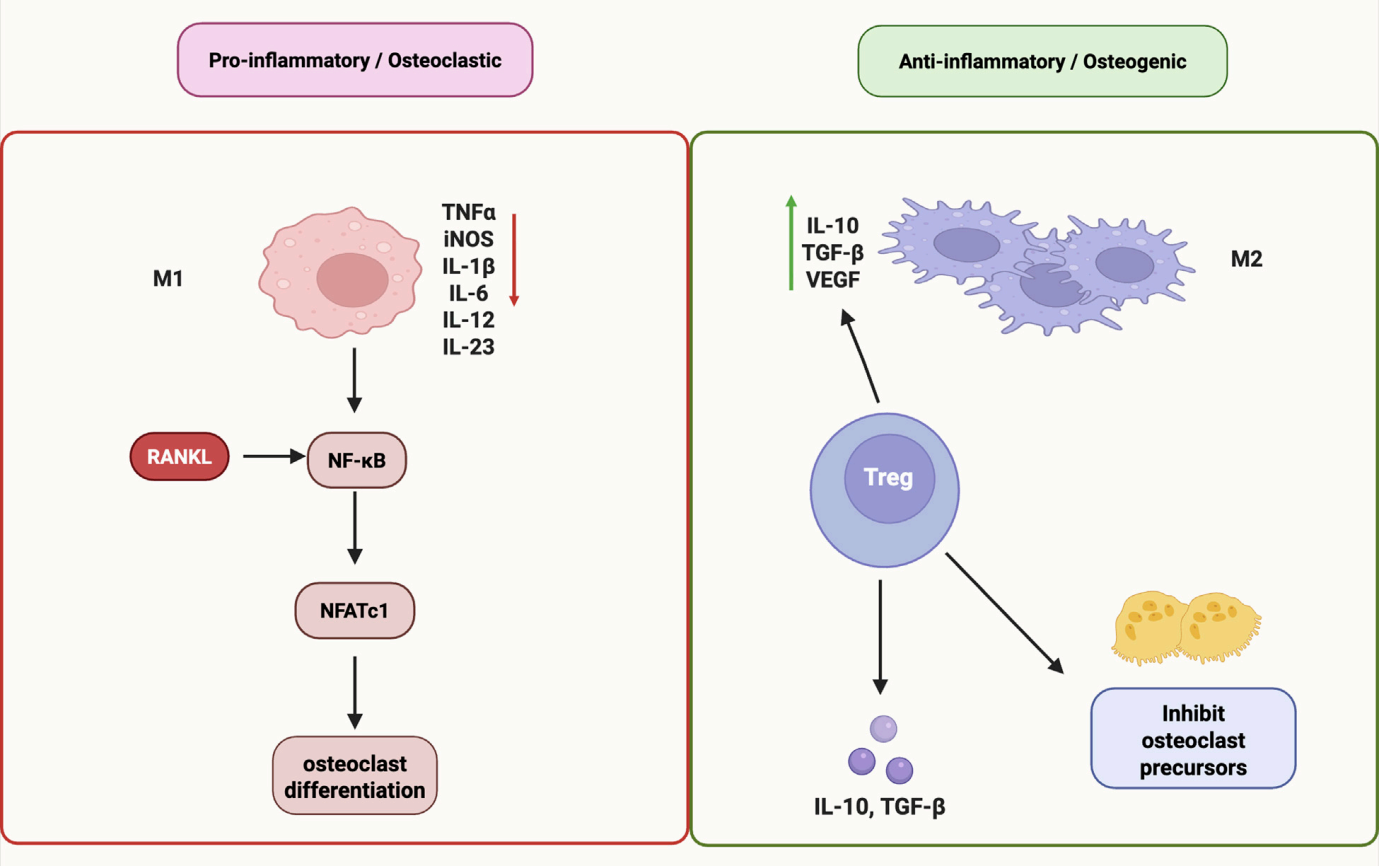
Immunomodulatory mechanisms of natural products in promoting bone regeneration. Natural products promote bone regeneration by modulating immune responses. They induce M2 macrophage polarization, enhance Treg cell differentiation, and suppress pro-inflammatory signaling via NF-κB and JAK–STAT pathways. These effects help balance osteoimmunity and support osteo-vascular repair. Abbreviations: M1, pro-inflammatory macrophage; M2, anti-inflammatory macrophage; RANKL, receptor activator of nuclear factor κB ligand; NF-κB, nuclear factor kappa-light-chain-enhancer of activated B cells; NFATc1, nuclear factor of activated T cells 1; TNFα, tumor necrosis factor alpha; iNOS, inducible nitric oxide synthase; IL, interleukin; IL-10, interleukin-10; IL-1β, interleukin-1 beta; IL-6, interleukin-6; IL-12, interleukin-12; IL-23, interleukin-23; TGF-β, transforming growth factor beta; VEGF, vascular endothelial growth factor; Treg, regulatory T cell.

Taken together, immune modulation—including M1-to-M2 macrophage repolarization, Treg induction, and NF-κB pathway inhibition—constitutes a crucial mechanism in maintaining the cellular equilibrium necessary for bone regeneration. In particular, restoring immune balance—particularly by promoting the polarization of macrophages toward the anti-inflammatory M2 phenotype and expanding the population of Tregs—has emerged as a promising strategy for enhancing bone regeneration. M2 macrophages support angiogenesis, secrete anti-inflammatory cytokines such as Interleukin-10 (IL-10) and Transforming growth factor-beta (TGF-β), and promote osteogenic. These findings underscore the capacity of natural products to modulate osteoimmune balance via M2 macrophage polarization, Treg cell induction, and inhibition of NF-κB/JAK-STAT signaling, ultimately promoting osteo-vascular regeneration (Figure 3).

#### 4.2.1 Macrophage M2 polarization enhances osteo-vascular regeneration

Natural products can effectively induce macrophage polarization toward the M2 phenotype, thereby mitigating chronic inflammation within the bone microenvironment, enhancing osteogenic differentiation of BMSCs, and indirectly promoting angiogenesis. This immunomodulatory mechanism has emerged as a key strategy for orchestrating bone regeneration through the immune–bone–vascular interface.

Resveratrol, a well-studied polyphenol isolated from the skins of *V. vinifera* L. (Vitaceae) and the roots of *P. cuspidatum* Siebold and Zucc. (Polygonaceae), has been shown to act as an autophagy activator that restores immune homeostasis in senescent macrophages, promoting their polarization toward the M2 phenotype. In rat models, resveratrol-loaded implant surfaces not only optimized the local immune microenvironment but also significantly enhanced osseointegration and neovascularization, highlighting its therapeutic relevance in biomaterial-based bone repair (Hang et al., 2022).

Picein, a phenolic glycoside isolated from the bark of *Picea abies* (L.) H. Karst. (Pinaceae), attenuates erastin-induced oxidative damage and ferroptosis by activating the Nrf2/HO-1/GPX4 signaling pathway, thereby enhancing the osteogenic potential of BMSCs and the angiogenic capacity of HUVECs. *In vivo*, picein promotes M2 polarization and accelerates the repair of critical-sized bone defects (Huang et al., 2024).

Catalpol, an iridoid glycoside isolated from the roots of Rehmannia glutinosa (Gaertn.) DC. (Orobanchaceae), exerts its osteo-regenerative effects by modulating the paracrine crosstalk between macrophages and BMSCs. It promotes M2 macrophage polarization, increases VEGF expression, and facilitates both ectopic bone formation and angiogenesis (Zhang et al., 2022).

Collectively, these studies suggest that M2 macrophage polarization is a predominant and well-supported mechanism by which natural products exert immunoregulatory functions in bone healing. Resveratrol and picein are notable for their *in vivo* evidence and mechanistic clarity, while catalpol represents a novel paradigm in modulating bone–immune–vascular interactions via paracrine signaling.

However, the current body of literature also presents several challenges. Inconsistencies in the selection and interpretation of M1/M2 polarization markers—such as Cluster of Differentiation 206 (CD206), Arginase-1 (Arg-1), or IL-10—limit cross-study comparability. Moreover, most studies fail to assess the temporal dynamics of macrophage polarization during the distinct phases of bone repair. Notably, the relative contribution of M2 macrophages to osteogenesis remains controversial across different pathological contexts, including diabetic osteoporosis, aging-related bone loss, and postmenopausal fractures. These discrepancies underscore the need for disease-specific immune stratification and standardized macrophage evaluation criteria in future research.

#### 4.2.2 Regulatory T cell induction modulates osteoimmune balance

Regulatory T cells play a pivotal role in maintaining osteoimmune homeostasis by secreting immunosuppressive cytokines such as IL-10 and TGF-β. These cytokines inhibit osteoclast differentiation and inflammatory activation while indirectly promoting osteoblast activity and bone formation. Within the context of bone regeneration, Treg-mediated immunomodulation has emerged as a promising strategy to mitigate chronic inflammation and restore bone remodeling balance.

ORI is one of the few natural small molecules known to promote the expansion of Treg populations. In a rat calvarial defect model, Yu et al. (2023) demonstrated that ORI significantly increases the proportion of Cluster of Differentiation 4 positive (CD4^+^), Cluster of Differentiation 25 positive (CD25^+^), Forkhead box P3 positive (Foxp3^+^) Treg cells while suppressing the expression of pro-inflammatory cytokines TNF-α and Interleukin-1 beta (IL-1β). Notably, ORI also activated the Wnt3a/β-catenin–VEGF signaling pathway, which synergistically enhanced trabecular bone quality, microvascular density, and local immune suppression, thereby facilitating coordinated osteogenic and angiogenic regeneration.

This evidence positions the “Treg-Wnt-VEGF” axis as a novel immunoregulatory paradigm in bone regeneration research, effectively establishing a mechanistic cascade from immune cell modulation to osteogenesis and neovascularization. Although literature in this area remains limited, Treg induction offers a high-value entry point for addressing complex inflammatory bone disorders—such as rheumatoid arthritis-associated bone erosion, diabetic osteoporosis, and peri-implant osteolysis—where conventional stem cell or anti-resorptive strategies may be insufficient.

However, several limitations should be acknowledged. Current studies lack dynamic tracking of Treg phenotype transitions during the course of bone repair, making it difficult to assess the temporal requirements of Treg-mediated effects. Furthermore, the spatial localization and tissue-specific function of Tregs within bone defects remain poorly defined. Most available data are derived from early-stage animal experiments, with limited long-term follow-up and few evaluations of hard tissue endpoints, such as BMD, biomechanical strength, or mature vascular networks.

In summary, the ability of natural products such as ORI to induce Tregs represents a mechanistically innovative and clinically relevant approach to osteoimmune modulation. Future studies should aim to integrate spatial–temporal mapping, lineage tracing, and multi-omics profiling to better delineate Treg dynamics during bone healing. Additionally, extending current findings to large animal models and longitudinal clinical trials will be essential for validating the translational potential of Treg-targeted osteoregenerative therapies.

#### 4.2.3 NF-κB/JAK-STAT inhibition suppresses pro-inflammatory signals

Persistent activation of inflammatory signaling pathways represents a major pathological driver of bone microenvironmental dysregulation. Natural products can attenuate excessive osteoclastogenesis and reduce osteoblast apoptosis by targeting classical inflammatory cascades, most notably the NF-κB and JAK-STAT pathways.

Curcumin has been extensively investigated for its dual antioxidant and anti-inflammatory properties. Mechanistically, curcumin enhances the expression of antioxidant enzymes—such as HO-1 and Superoxide dismutase (SOD)—via activation of the GSK3β–Nrf2 axis, while simultaneously inhibiting M1 macrophage polarization and reducing the secretion of pro-inflammatory cytokines such as TNF-α and IL-6 (Li X et al., 2020). In osteoblasts, curcumin further suppresses inflammation-induced injury by downregulating the JAK1/2–STAT1 signaling cascade, thereby preserving osteogenic capacity under inflammatory stress (Jin et al., 2020). These multifaceted regulatory effects have been validated across diverse experimental models of osteoporosis, bone defect, and inflammatory bone loss.

Despite its well-defined mechanisms, curcumin’s low bioavailability and rapid metabolic clearance limit its clinical efficacy, prompting ongoing efforts to improve its formulation through nanocarriers, prodrug strategies, or bioenhancers. Moreover, the role of NF-κB in bone regeneration is complex and context-dependent. While its suppression alleviates chronic inflammation, complete inhibition of NF-κB signaling has been shown to impair osteoblast proliferation, migration, and early differentiation, suggesting that its therapeutic targeting must be finely tuned in terms of dosage, timing, and pathway specificity.

### 4.3 Oxidative stress and ferroptosis regulation: NRF2/GPX4 axis and mitochondrial homeostasis

Beyond immune regulation, oxidative stress represents a pivotal non-cellular mechanism contributing to the disruption of bone microenvironmental homeostasis. Oxidative stress arises from an imbalance between the excessive generation of reactive species—including ROS and reactive nitrogen species (RNS)—and the cellular antioxidant defense capacity, resulting in redox disequilibrium and subsequent molecular damage. This redox imbalance initiates a cascade of deleterious effects, including Deoxyribonucleic acid (DNA) strand breaks, protein carbonylation, and lipid peroxidation, ultimately impairing cellular viability and function (Juan et al., 2021). In osteogenic contexts, oxidative stress activates signaling pathways such as NF-κB and MAPK, leading to inflammatory responses, osteoblast apoptosis, cellular senescence, and, in severe cases, mitochondria-dependent forms of programmed cell death (Mukherjee et al., 2022; Shi et al., 2023). Elevated ROS levels within the bone niche have been shown to impair osteoblast function, degrade ECM components, inhibit osteogenic differentiation of BMSCs, and sustain M1-polarized macrophages, thereby exacerbating skeletal injury and impairing tissue repair (Ye et al., 2023).

Importantly, ROS exhibit a context-dependent dual role in bone biology. At early stages of bone injury or remodeling, moderate ROS levels serve as signaling mediators that facilitate cell migration, phagocytosis, and tissue regeneration (Li et al., 2021). However, when ROS levels remain chronically elevated and antioxidant enzyme systems—including SOD, Catalase (CAT), and GPX—are overwhelmed, redox toxicity ensues. This leads to mitochondrial dysfunction, osteoblast apoptosis, and overall degradation of bone cell homeostasis (Marques-Carvalho et al., 2023). Therefore, targeted modulation of local oxidative stress and restoration of antioxidant defenses are critical strategies to enhance bone regeneration and maintain microenvironmental equilibrium.

Recent investigations have increasingly focused on the activation of canonical antioxidant pathways, particularly the Nrf2/HO-1 and Nrf2/GPX4 axes, to mitigate oxidative injury and suppress ferroptosis—a non-apoptotic, iron-dependent form of regulated cell death characterized by lipid peroxidation. Several natural products derived from TCM have exhibited promising dual activity in promoting both antioxidant defense and osteogenesis, though their mechanisms vary.

Among these, punicalagin, an ellagitannin isolated from the peels of *Punica granatum* L. (Lythraceae); naringenin, a flavanone predominantly found in the fruits of *Citrus paradisi* Macfad. (Rutaceae); and geniposide, an iridoid glycoside extracted from the fruits of *Gardenia jasminoides* J. Ellis (Rubiaceae), are representative agents that activate the Nrf2/HO-1 axis. Punicalagin significantly upregulates Nrf2/HO-1 expression, alleviates oxidative stress in BMSCs, and enhances their osteogenic differentiation. Additionally, it promotes endothelial cell migration and angiogenic factor expression, and induces M2 macrophage polarization, thereby contributing to a pro-regenerative immune milieu (Huang et al., 2023). Geniposide, an iridoid glycoside isolated from *G. jasminoides*, activates the Nrf2/HO-1/NAD(P)H quinone dehydrogenase 1 (NQO1) signaling cascade, reducing cadmium-induced ROS accumulation and preventing osteoblast apoptosis through enhanced antioxidant enzyme expression (He et al., 2019).

More recently, products with potential to inhibit ferroptosis—such as crocin and cardamonin—have garnered attention for their multifaceted regulatory roles. Crocin, a carotenoid glycoside isolated from the stigmas of *Crocus sativus* L. (Iridaceae), activates the Nrf2/GPX4 axis, reduces intracellular ROS levels, enhances antioxidant capacity, and concurrently suppresses ferroptosis. In both *in vitro* and *in vivo* models, it has been reported to facilitate osteoblastogenesis and angiogenesis (Hong et al., 2025). Cardamonin, a chalcone-type flavonoid isolated from the fruits of *Alpinia katsumadai* Hayata (Zingiberaceae), modulates the HIF-1α/ROS axis, mitigates oxidative injury in iron-overload murine models, and promotes osteogenic differentiation (Chen et al., 2024).

Additional products including curculigoside, a phenolic glycoside isolated from the rhizomes of *Curculigo orchioides* Gaertn. (Hypoxidaceae), and apocynin, a methoxy-substituted catechol derivative obtained from the roots of *Apocynum cannabinum* L. (Apocynaceae), have demonstrated combined antioxidant and anti-inflammatory properties. Curculigoside exerts its effects by activating Nrf2 while suppressing NADPH oxidase (NOX) and NF-κB signaling pathways (Liu et al., 2021). Apocynin has been shown to protect mitochondrial function and reduce apoptosis in dexamethasone-induced osteoblast injury models, ameliorating glucocorticoid-associated osteopathy (Zhang et al., 2023). Neobavaisoflavone, an isoflavone compound isolated from the seeds of *Psoralea corylifolia* L. (Fabaceae), alleviates oxidative stress and enhances osteogenic differentiation via upregulation of Long non-coding RNA colorectal neoplasia differentially expressed (lncRNA-CRNDE) and activation of the Nrf2/HO-1 pathway (Zhu P et al., 2021).

Collectively, the Nrf2/HO-1 and GPX4 pathways constitute well-established molecular targets for antioxidant-mediated bone protection, with products such as punicalagin, naringenin, and geniposide demonstrating consistent efficacy across experimental platforms. In contrast, crocin and cardamonin introduce innovative mechanistic dimensions involving ferroptosis regulation and hypoxia–oxidative stress signaling; however, current supporting evidence is limited to acute experimental models. Curculigoside and apocynin offer integrated anti-inflammatory and antioxidative strategies but require further validation due to inter-study heterogeneity in methodologies and endpoints.

**FIGURE 4:**
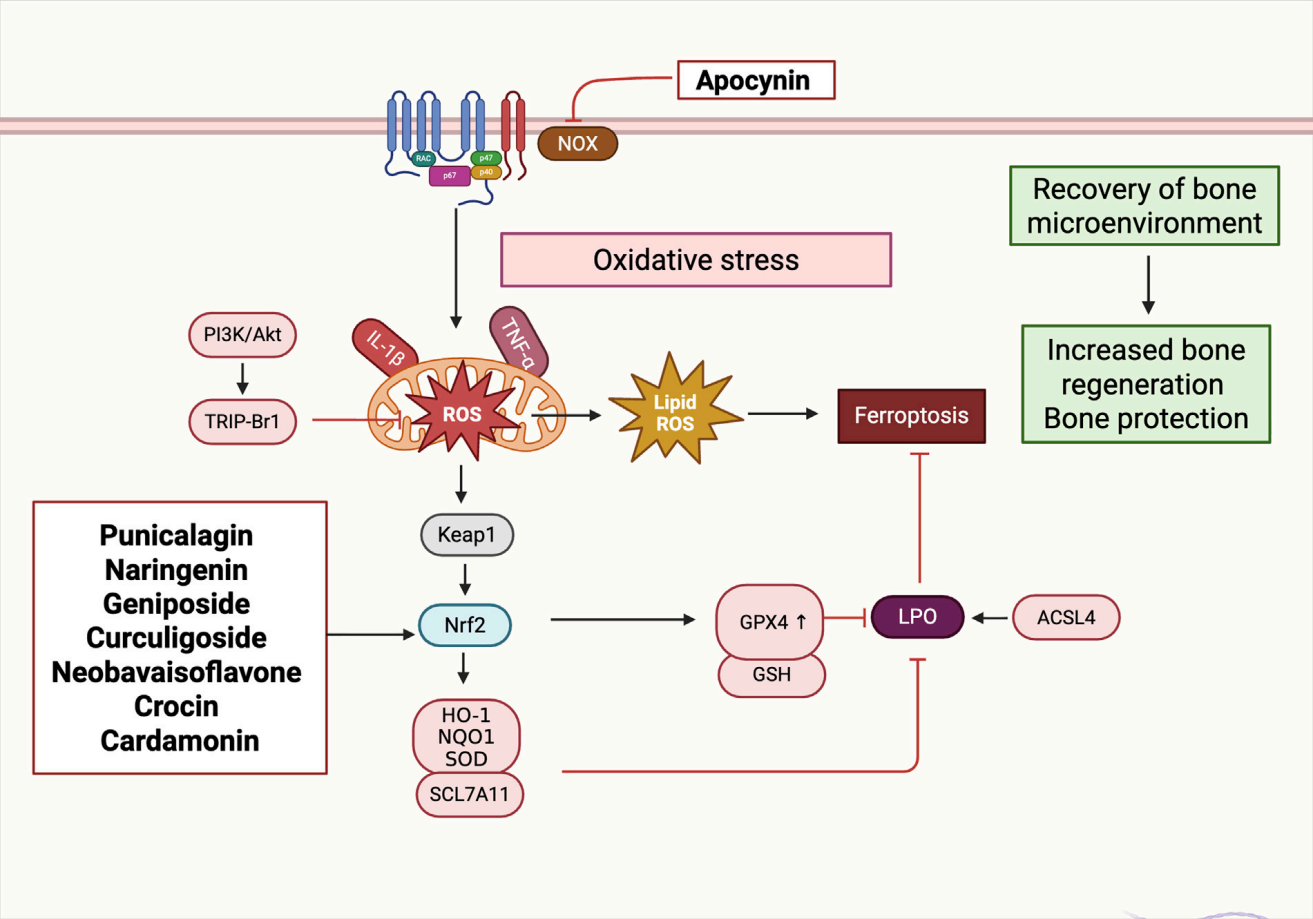
Natural Products modulate oxidative stress and ferroptosis via the Nrf2/GPX4 signaling axis to restore bone homeostasis. Natural products activate the Nrf2/GPX4 axis to counter oxidative stress and inhibit ferroptosis during bone remodeling. By reducing ROS and lipid peroxidation, these agents help restore the bone microenvironment and promote regeneration. Abbreviations: NOX, NADPH oxidase; PI3K, phosphoinositide 3-kinase; Akt, protein kinase B; TRIP-Br1, transcriptional regulator interacting with the PHD-bromodomain 1; IL-1β, interleukin-1 beta; TNFα, tumor necrosis factor alpha; ROS, reactive oxygen species; Lipid ROS, lipid-derived reactive oxygen species; Keap1, Kelch-like ECH-associated protein 1; Nrf2, nuclear factor erythroid 2–related factor 2; HO-1, heme oxygenase-1; NQO1, NAD(P)H quinone oxidoreductase 1; SOD, superoxide dismutase; SLC7A11, solute carrier family 7 member 11; GPX4, glutathione peroxidase 4; GSH, glutathione; LPO, lipid peroxidation; ACSL4, acyl-CoA synthetase long-chain family member 4.

While evidence continues to accumulate, most investigations emphasize the cytotoxic effects of ROS while overlooking their physiological signaling roles during early phases of bone repair. Moreover, standardized methodologies for real-time ROS detection, ferroptosis quantification, and long-term assessment in clinically relevant bone disease models are lacking. Future research should prioritize the establishment of robust detection platforms and longitudinal studies to fully elucidate the translational potential of antioxidant and ferroptosis-modulating phytochemicals in bone regeneration. These findings highlight the role of natural products in alleviating oxidative stress and ferroptosis via the Nrf2/GPX4 pathway, thereby restoring mitochondrial function and supporting bone homeostasis (Figure 4).

### 4.4 Gut microbiota-mediated regulation of bone remodeling by natural products

The gut–bone axis has recently emerged as a critical regulatory mechanism in bone metabolism, highlighting the systemic influence of gut microbiota on skeletal homeostasis. Gut microbiota modulate the balance between osteoblastogenesis and osteoclastogenesis through multiple interconnected mechanisms, including the regulation of nutrient absorption, reinforcement of intestinal barrier integrity, modulation of immune cell subsets, and production of bioactive microbial metabolites. A healthy gut microbiota profile enhances the expression of tight junction proteins (e.g., occludin, Zonula occludens-1 (ZO-1)), reduces intestinal permeability, and limits the systemic translocation of pro-inflammatory mediators such as lipopolysaccharide (LPS), thereby attenuating chronic low-grade inflammation associated with bone loss (Luo et al., 2025).

In addition, gut microbial communities contribute to bone immune homeostasis by modulating the differentiation and activity of T helper 17 (Th17) cells and Tregs, thus preventing aberrant osteoclast activation (Guo et al., 2023; Qi et al., 2024). Notably, short-chain fatty acids (SCFAs)—key microbial metabolites such as butyrate and propionate—play a pivotal role by activating G protein-coupled receptors (GPCRs) and inhibiting the RANKL–NF-κB signaling axis. This leads to the downregulation of critical osteoclastogenic mediators, including TRAF6 and NFATc1, ultimately suppressing bone resorption (Lucas et al., 2018).

Emerging data also implicate gut-derived hormones—such as glucagon-like peptide-1 (GLP-1), Peptide YY (PYY), and gastric inhibitory polypeptide (GIP)—in the neuroendocrine regulation of bone remodeling. These hormones directly influence osteoblast and osteoclast function and indirectly improve the bone microenvironment by modulating systemic metabolic status (Hansen et al., 2023; Skov-Jeppesen et al., 2024).

Natural products have shown significant potential to modulate the gut microbiota and, consequently, influence bone metabolism via the gut–bone axis. Several metabolites selectively promote the growth of probiotic bacteria, enhance microbial diversity, increase SCFA production, and restore immune homeostasis, thus offering promising therapeutic strategies for metabolic bone diseases. For instance, quercetin was shown to increase the abundance of beneficial gut microbes, elevate SCFA levels, suppress inflammatory cytokines, and improve both trabecular architecture and BMD in OVX rat models (Feng et al., 2024). Similarly, puerarin, an isoflavone compound isolated from the roots of *Pueraria lobata* (Willd.) Ohwi (Fabaceae), demonstrated anti-osteoporotic effects in OVX rats through microbiota-dependent mechanisms, including improved mucosal barrier integrity, reduced systemic inflammation, and modulation of microbial metabolism—particularly pathways involved in amino acid metabolism, LPS biosynthesis, and butyrate production (Li X et al., 2020).

Further evidence from Li et al. suggests that TFRD promote bone formation in OVX rats by reshaping the gut microbial composition and enhancing SCFA biosynthesis. In particular, the enrichment of *Akkermansia muciniphila* was positively correlated with improved bone mass (Li Q et al., 2025).

Gut microbiota–mediated bone remodeling by natural products offers unique advantages, including multi-target regulation, metabolism-dependent activity, and favorable safety profiles. Without directly acting on bone cells, these metabolites influence bone homeostasis through microbial metabolites, immune signaling, and hormonal pathways. Additionally, many phytochemicals undergo microbial biotransformation into more bioactive metabolites, potentially enhancing efficacy while minimizing systemic toxicity. These characteristics confer enhanced tissue selectivity and support long-term intervention potential.

**FIGURE 5:**
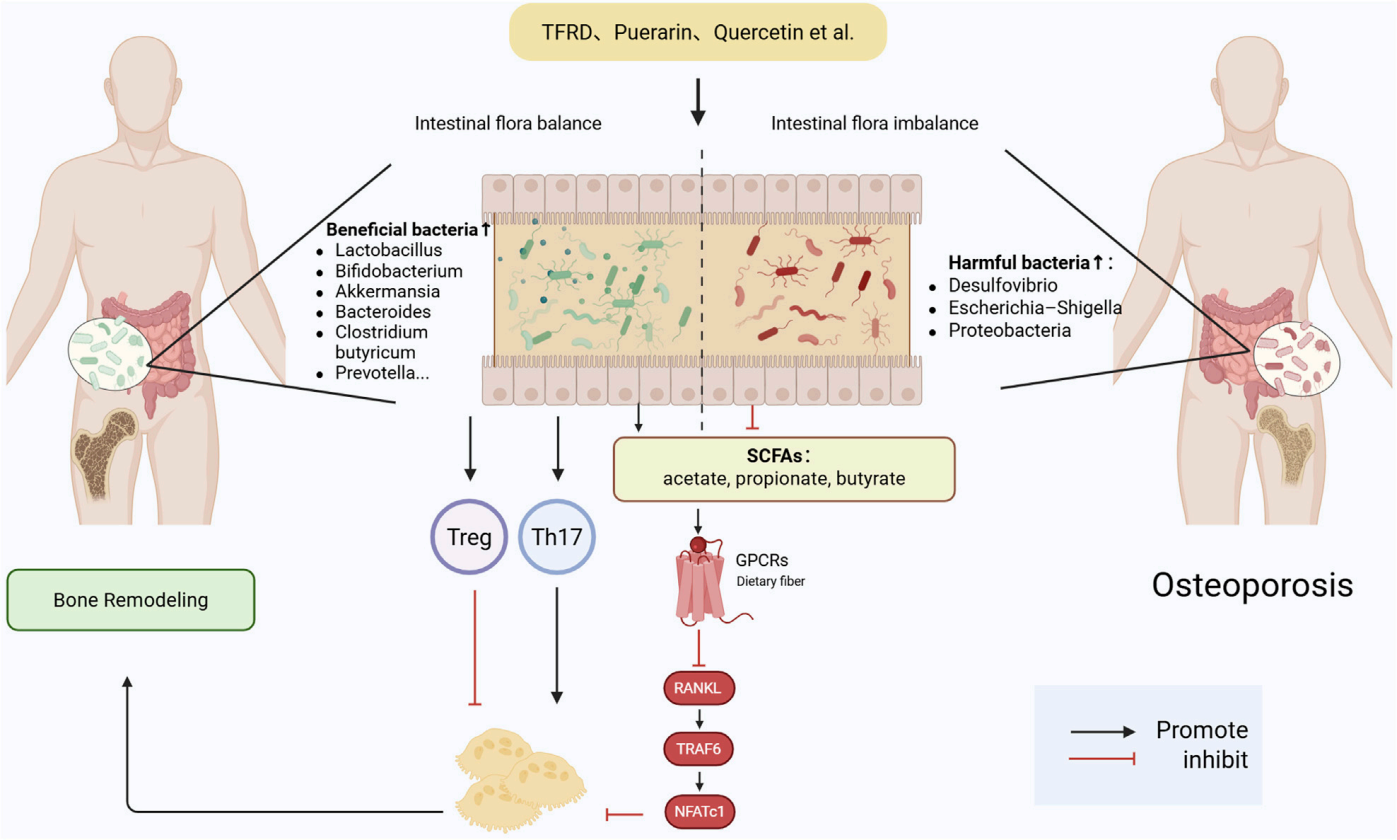
Gut microbiota-mediated mechanisms by which natural products regulate bone remodeling. Natural products regulate bone remodeling via the gut microbiota by enhancing SCFA production, modulating GPCR and RANKL–TRAF6–NFATc1 pathways, and balancing immune responses through Th17/Treg regulation and gut hormones. Abbreviations: Wnt, wingless-related integration site; LRP, low-density lipoprotein receptor-related protein; BMP, bone morphogenetic protein; SMAD, mothers against decapentaplegic homolog; PI3K, phosphoinositide 3-kinase; Akt, protein kinase B; MAPK, mitogen-activated protein kinase; ERK, extracellular signal-regulated kinase; AP-1, activator protein 1; NF-κB, nuclear factor kappa-light-chain-enhancer of activated B cells; TRAF6, TNF receptor-associated factor 6; RANK, receptor activator of nuclear factor κB; RANKL, receptor activator of nuclear factor κB ligand; OPG, osteoprotegerin; NFATc1, nuclear factor of activated T cells 1; IL, interleukin; IL-17RC, interleukin-17 receptor C; IL-17RA, interleukin-17 receptor A; CCL2, C-C motif chemokine ligand 2; CCR2, C-C motif chemokine receptor 2; CXCL12, C-X-C motif chemokine ligand 12; CXCR4, C-X-C motif chemokine receptor 4; LBP, lipopolysaccharide-binding protein; Bcl-2, B-cell lymphoma 2; Bax, Bcl-2-associated X protein; MOMP, mitochondrial outer membrane permeabilization; Cyt c, cytochrome c; Caspase-9, cysteine-aspartic acid protease 9; Caspase-3, cysteine-aspartic acid protease 3; ALP, alkaline phosphatase; COL1, collagen type I; Runx2, runt-related transcription factor 2; Osterix, transcription factor Sp7; TRAP, tartrate-resistant acid phosphatase; DC-STAMP, dendritic cell-specific transmembrane protein.

Nevertheless, this regulatory strategy also presents notable limitations. First, interindividual variability in microbiota composition—shaped by host genetics, age, and diet—leads to inconsistent therapeutic outcomes (Schupack et al., 2022). Second, poor oral bioavailability and unstable pharmacokinetics remain major obstacles for many natural products. Third, the complexity of microbial metabolism poses challenges for mechanistic prediction, making it difficult to establish causal links between specific microbial taxa and bone-related outcomes (Wang Y et al., 2023). Therefore, while the gut–bone axis represents a promising avenue for bone regeneration therapy, further mechanistic studies are required to elucidate host–microbiota–metabolite interactions, identify key microbial targets, and optimize combinatorial treatment strategies. An overview of the representative prodcuts, microbial targets, and proposed mechanisms is summarized in Table 2, while the synergistic effects of natural products and gut microbiota on the bone microenvironment are illustrated in Figure 5.

**TABLE 2:** Summary of natural products modulating bone metabolism via the gut microbiota–SCFAs axis.

Compound	Botanical source	Extract type/Solvent	Type of model	Working concentration	Microbiota modulation	SCFAs change	Proposed axis/Mechanism	Research stage	References
Berberine	an isoquinoline alkaloid isolated from the rhizomes of *Coptis chinensis* Franch. (Ranunculaceae)	Purified monomer	C57BL/6 ligature-induced periodontitis mouse model	50 mg/kg/day by gavage for 10 days (*in vivo*)	↑ Firmicutes, Bacteroidetes, Muribaculaceae, Alistipes; ↓ Proteobacteria, Campylobacterota; restored microbial diversity	↑ Acetate, propionate, butyrate in feces (analyzed via GC-MS)	“Gut microbiota–SCFAs–immune regulation” axis; Berberine modulates gut microbiota to increase SCFAs, reduce IL-6 and TNF-α, inhibit osteoclastogenesis	*In vivo*	Jia et al. (2019)
Quercetin	a flavonol widely distributed in the flowers of *Sophora japonica* L. (Fabaceae), the bulbs of *Allium cepa* L. (Amaryllidaceae), and the leaves of *Camellia sinensis* (L.) Kuntze (Theaceae)	Purified monomer	OVX rat model with/without ABX (antibiotic) and FMT (fecal microbiota transplantation)	50 mg/kg/day by gavage for 6 weeks (*in vivo*)	↑ Lactobacillales, Prevotellaceae, Blautia; ↓ Desulfobacterota, Erysipelotrichales, Romboutsia, Butyricoccaceae	↑ SCFAs (e.g., acetate, propionate, butyrate) in feces	Intestinal flora → SCFAs → ↓ LPS, IL-1β, TNF-α → ↓ colonic permeability (↑ ZO-1, Occludin) → Bone protection	*In vivo*	Feng et al. (2024)
Dihydromyricetin	a flavanonol isolated from the leaves of *Ampelopsis grossedentata* (Hand.-Mazz.) W.T. Wang (Vitaceae)	Purified monomer	OVX mouse model	100 mg/kg/day by gavage for 6 weeks (*in vivo*)	↑ Akkermansia, Prevotellaceae, Lachnospiraceae; ↓ *Helicobacter*, Desulfovibrio	↑ Acetate, Propionate, Butyrate	DHM → gut microbiota/SCFAs → ↓ inflammation, ↑ osteogenesis	*In vivo*	Xu et al. (2024)
Anthocyanins	polyphenolic pigments widely distributed in the fruits of *Vaccinium myrtillus* L. (Ericaceae) and *Vitis vinifera* L. (Vitaceae)	Purified monomer	High-fat diet (HFD)-induced metabolic syndrome mouse model (C57BL/6)	150 mg/kg/day by gavage for 12 weeks (*in vivo*)	↑ Bifidobacterium, Akkermansia, Allobaculum; ↓ Desulfovibrio, Oscillibacter	↑ Total SCFAs, especially acetic acid and butyric acid	“Gut microbiota–SCFAs–inflammation–metabolism” axis	*In vivo*	Du et al. (2024)
Baicalin	a flavone glycoside isolated from the roots of *Scutellaria baicalensis* Georgi (Lamiaceae)	Purified monomer	Aged ligature-induced periodontitis mouse model (C57BL/6, 18 months old)	50 mg/kg/day, administered orally for 14 days	↑*Lactobacillus*, ↓Desulfovibrio, ↓Escherichia–Shigella; modulated oral and gut microbial composition	↑Acetate, propionate, and butyrate levels in feces	Regulates microbiota–SCFAs–inflammation axis to inhibit bone resorption	*In vivo*	Hu et al. (2024)
Icariin	a prenylated flavonol glycoside isolated from the aerial parts of *Epimedium brevicornum* Maxim. (Berberidaceae)	Purified monomer	OVX rat model	100 mg/kg/day by gavage for 12 weeks (*in vivo*)	↑*Lactobacillus*, Bifidobacterium, *Bacteroides*; ↓*Clostridium*, Desulfovibrio	↑acetic acid, Propionic acid, Butyric acid in feces	Icariin → Gut microbiota modulation → SCFAs ↑ → Systemic metabolite shift (e.g., bile acids, amino acids) → ↓bone resorption and ↑bone formation	*In vivo*	Wang et al. (2022)
Lignan-rich fraction	isolated from the stems and bark of *Sambucus williamsii* Hance (Adoxaceae)	Ethanol (60%) reflux extract, HP-20 resin fraction (50% EtOH eluate)	OVX rat model	100 mg/kg/day by gavage for 12 weeks (*in vivo*)	↑ Akkermansia, *Bacteroides*, Rikenellaceae_RC9_gut_group ↓ Lachnospiraceae_NK4A136_group, Desulfovibrionaceae	none	“Gut microbiota – metabolism – metabolic health” axis	*In vivo*	Xiao et al. (2021)
4-Hydroxyphenylacetic acid	a microbial-derived phenolic acid metabolite produced during gut fermentation of dietary polyphenols	Microbial metabolite (non-extract)	BMMs from 6-week-old C57BL/6 mice induced by RANKL	10/20/40 μM of 4-HPAA for 7 days	none	none	4-HPAA → ↓ROS production → ↓NFATc1 activation → ↓osteoclastogenesis	*In vitro*	Zhang et al. (2024)
Curcumin	a diarylheptanoid polyphenol derived from the rhizomes of *Curcuma longa* L. (Zingiberaceae)	Purified monomer	Glucocorticoid-induced osteoporosis (GIOP) mouse model	100 mg/kg/day by gavage for 4 weeks (*in vivo*)	↑ Beneficial genera (*Lactobacillus*, Ruminococcus), ↓ Pathogenic taxa (Escherichia-Shigella)	↑ acetic acid, butyric acid, valeric acid in fecal samples	Gut microbiota–SCFAs–metabolome–bone metabolism axis	*In vivo*	Li S et al. (2025)
Black tea enriched with Polygonatum sibiricum polysaccharides (PSP)	extracted from the rhizomes of *Polygonatum sibiricum* Redouté (Asparagaceae)	Hot-water extract enriched with polysaccharides (compound black tea beverage, CBT)	OVX mouse model	20 mg/kg, administered orally for 12 weeks	↓ Firmicutes/Bacteroidetes ratio; ↑ beneficial microbes (e.g., *Lactobacillus*); ↓ *Helicobacter*	↑ Total SCFAs (acetic acid, propionic acid, butyric acid) in feces	“Gut microbiota–SCFAs–immune response–bone” axis; PSP improves bone loss via modulating gut microbiota, enhancing SCFAs, and reducing Th17/Treg imbalance and inflammation	*In vivo*	Gao et al. (2025)

### 4.5 From bench to bedside: translational barriers of botanical drug

Despite extensive preclinical evidence supporting the multi-target and multi-pathway activities of various natural products in regulating oxidative stress, immune homeostasis, osteogenic differentiation, and angiogenesis, their translation from mechanistic validation to clinical application remains significantly hindered by several challenges.

Numerous plant-derived bioactive products have shown potent regulatory effects on osteo–angiogenic coupling *in vitro* and *in vivo*. Among them, flavonoids (e.g., icariin), glycosides and saponins (e.g., astragaloside IV, ginsenoside Rg1, compound K), and phenolics (e.g., polydatin, curcumin) are the most extensively studied categories (Zheng et al., 2024a; Wang et al., 2021; Chen W et al., 2022; Ding et al., 2022; Zhou et al., 2025). These products consistently activate conserved signaling pathways—particularly Wnt/β-catenin, PI3K/AKT, and VEGF/Notch axis—facilitating coordinated promotion of osteogenesis and angiogenesis.

Several products also exert effects through less conventional mechanisms. For instance, catalpol demonstrates immunomodulatory properties, while SAR exhibit regulatory effects on ferroptosis pathways (F et al., 2025). Cross-comparison of mechanistic studies reveals frequent enrichment of canonical signaling axes including Wnt/β-catenin, PI3K/AKT, VEGF/Notch, PDGF-BB, and HIF-1α/VEGF. Among these, the Wnt/β-catenin pathway appears as the most commonly targeted, serving as a converging node for flavonoids, saponins, and phenolics (Zhu Z et al., 2021). This suggests it may represent a core regulatory hub in mediating osteo-angiogenic coupling.Likewise, the PI3K/AKT and VEGF axes have been repeatedly validated in studies involving *A. membranaceus*, *P. notoginseng*, *P. ginseng*, and various flavonoid-rich extracts, highlighting their functional robustness across diverse phytochemical classes (Shan et al., 2023; Shen et al., 2022). However, despite this mechanistic consistency at the pathway level, a number of discrepancies and knowledge gaps remain.

For example, curcumin has been reported to restore VEGF expression and enhance angiogenesis in diabetic bone defect models, yet fails to exhibit similar pro-angiogenic effects in inflammation-driven environments—underscoring its strong microenvironmental dependency. Similarly, conflicting findings exist regarding echinacoside’s anti-osteoclastic activity: while some studies report suppression of MMP9 and CTSK, others fail to observe consistent inhibitory effects on osteoclast differentiation or function.

Pharmacokinetic limitations remain one of the most formidable challenges impeding the clinical translation of natural products. Most bioactive metabolites derived from medicinal plants exhibit low oral bioavailability, limited tissue exposure, and rapid metabolic clearance. For instance, ICA has an oral bioavailability of approximately 12% (F_rel ∼12%), primarily constrained by poor intestinal membrane permeability and extensive first-pass hepatic metabolism (Li et al., 2013). Animal studies further reveal that ICA is rapidly biotransformed into its active metabolite icariside II, which demonstrates superior *in vivo* exposure—with Maximum plasma concentration (C_max) and Area under the plasma concentration–time curve (AUC) values 3.8- and 13-fold higher than the parent metabolite, respectively (Cheng et al., 2015). To address these limitations, advanced formulation strategies such as nanosuspensions and cyclodextrin inclusion complexes have been explored, achieving 2- to 4-fold improvements in systemic bioavailability (Ding et al., 2025).

Although these enhancements show promise, the translation into human studies remains limited. A 24-month randomized, double-blind, placebo-controlled trial confirmed that icariin-containing formulations are both effective and well-tolerated in preventing postmenopausal OP (Zhang G et al., 2007). In addition, a recent pharmacokinetic trial in postmenopausal women validated the product’s safety and preliminary systemic activity (Yong et al., 2021). However, most natural flavonoid glycosides and saponins remain in the preclinical stage, with pharmacokinetic data restricted to rodent or canine models. Their oral bioavailability is strikingly low—only 7.4% in dogs and 3.7% in rats (Stępnik et al., 2025; Zhang Q et al., 2007). Ginsenoside Rg1 similarly demonstrates limited absorption in rats (∼18.4%), and both *in vitro* Caco-2 cell transport models and *in vivo* studies confirm poor membrane permeability (∼3.3%) (Xu et al., 2003). These findings underscore the difficulty of achieving therapeutic plasma concentrations, thereby necessitating further optimization through delivery technologies.

Other representative products face similar limitations. Curcumin and ORI display extremely poor oral bioavailability and require encapsulation in liposomes or inclusion into cyclodextrins to enhance systemic exposure (Xu et al., 2006; Yakubu and Pandey, 2024). Although POL exhibits slightly better absorption than curcumin, its rapid metabolism limits its therapeutic window. For macromolecular polysaccharides such as APS, issues such as structural heterogeneity, poor oral absorption, and batch-to-batch inconsistency complicate quality control and clinical development (Wang et al., 2017). In addition, novel molecules like salvianolic acid R offer strong mechanistic novelty but lack systematic toxicological evaluation and multi-organ safety profiling (Mustafa et al., 2022). Currently, clinical trials in this field remain sparse, with only a few products such as catalpol having preliminary human safety data in the context of osteoporosis. Notably, high-quality clinical studies focusing on osteogenesis–angiogenesis coupling as a primary efficacy endpoint are still absent.Standardization and regulatory barriers further compound these challenges.

Although China has established foundational frameworks such as the *Standards for Chinese Medicinal Materials* and *Technical Guidelines for New Traditional Chinese Medicines*, these are primarily tailored for decoction pieces or complex multi-herb formulations (Leong et al., 2020). Comprehensive evaluation systems for near-pharmaceutical-grade monomers or purified extracts remain underdeveloped—particularly regarding bioequivalence testing, pharmacokinetics, long-term stability, and manufacturing reproducibility (Yan et al., 2018). For example, standardized phytochemicals such as punicalagin and crocin often display considerable variability in active content due to differences in plant source, cultivation environment, harvesting time, and extraction methodology (Ali et al., 2022; Cerdá et al., 2003). Such inconsistencies hinder consistent pharmacological efficacy and create regulatory obstacles in meeting clinical-grade quality standards.International pharmacopoeias—such as those issued by the World Health Organization (WHO), the United States Pharmacopeia, and the European Pharmacopoeia (Ph. Eur.)—have adopted analytical techniques such as chromatographic fingerprinting and marker product quantification to enhance quality control, most current standards remain restricted to single-component quantification and lack the capacity to comprehensively characterize the synergistic interactions inherent in multicomponent herbal formulations (Noviana et al., 2022). Consequently, these approaches fall short in ensuring batch-to-batch consistency and pharmacological reproducibility.

Moreover, regulatory frameworks in many developing countries have yet to mandate the use of chromatographic fingerprinting as a compulsory quality control measure, nor have they implemented evaluation criteria that align with evidence-based clinical standards. These regulatory and methodological gaps continue to impede the international registration, standardization, and clinical translation of botanical drug.

To address these limitations, future efforts should prioritize the improvement of source-level quality assurance for plant-derived bioactive products, including the standardization of raw material sourcing, processing methods, and botanical authentication. Concurrently, the development and refinement of multicomponent quantitative fingerprinting strategies capable of capturing both chemical complexity and pharmacological consistency are urgently needed. In addition, the integration of pharmacokinetic–pharmacodynamic correlation studies, particularly in translational models, will be essential to substantiate therapeutic efficacy. These efforts should be aligned with globally recognized Chemistry, Manufacturing, and Controls guidelines to ensure quality traceability and regulatory compliance.

Importantly, incorporating standardized, pharmacologically characterized botanical drugs into national pharmacopoeias or industry-recognized monographs would establish a robust foundation for subsequent clinical validation and regulatory approval. Ultimately, the reliable application of plant-derived natural metabolites in clinical settings requires the achievement of clearly defined chemical composition, consistent product quality, and traceable pharmacokinetic profiles. Only under such conditions can their multifaceted biological effects—such as antioxidative stress modulation, osteoblastogenesis potentiation, and immunoregulatory activity—be rigorously validated and reproducibly translated into evidence-based therapeutic strategies for metabolic bone disorders.

## 5 Advances in the synergistic promotion of bone regeneration by natural bioactive products and biomaterials

To address the multifactorial pathological microenvironment characteristic of osteoporosis-associated bone defects, a synergistic therapeutic paradigm combining natural bioactive products with functional biomaterials has emerged as a promising strategy. This integrative approach aims to achieve spatially localized, temporally controlled, and sustained release of therapeutic agents, thereby enhancing multi-targeted biological effects—including the potentiation of osteoblastogenesis, stimulation of angiogenesis, attenuation of inflammatory cascades, and mitigation of oxidative stress—to collectively promote bone regeneration.

When administered alone, many natural products are limited by inherent physicochemical constraints, such as poor aqueous solubility, low oral bioavailability, and rapid systemic clearance. In contrast, advanced biomaterial-based platforms—including biodegradable polymers, mesoporous bioactive glasses (BG), and stimuli-responsive nanocarriers—have demonstrated the ability to improve product stability, facilitate targeted delivery, and enable controlled drug release kinetics.

Biomaterials used for bone tissue engineering generally include biodegradable polymers, bioceramics, bioactive glasses, and composite materials that integrate both components.Polymers such as poly (lactic-co-glycolic acid) (PLGA) and polycaprolactone (PCL) offer controllable degradability, flexibility, and ease of processing, making them ideal for drug encapsulation and sustained release.Bioceramics, including hydroxyapatite (HA) and β-tricalcium phosphate (β-TCP), exhibit excellent osteoconductivity and chemical similarity to natural bone minerals, facilitating cell adhesion and bone integration. BG provide a unique capability to release therapeutic ions (e.g., Ca^2+^, Si^4+^, Sr^2+^), which enhance osteogenesis and angiogenesis.Combining these materials in hybrid scaffolds allows simultaneous optimization of mechanical strength, biodegradability, and biological activity, forming a versatile platform for natural compound delivery and bone regeneration (Bose and Tarafder, 2012; Dec et al., 2022).

**FIGURE 6:**
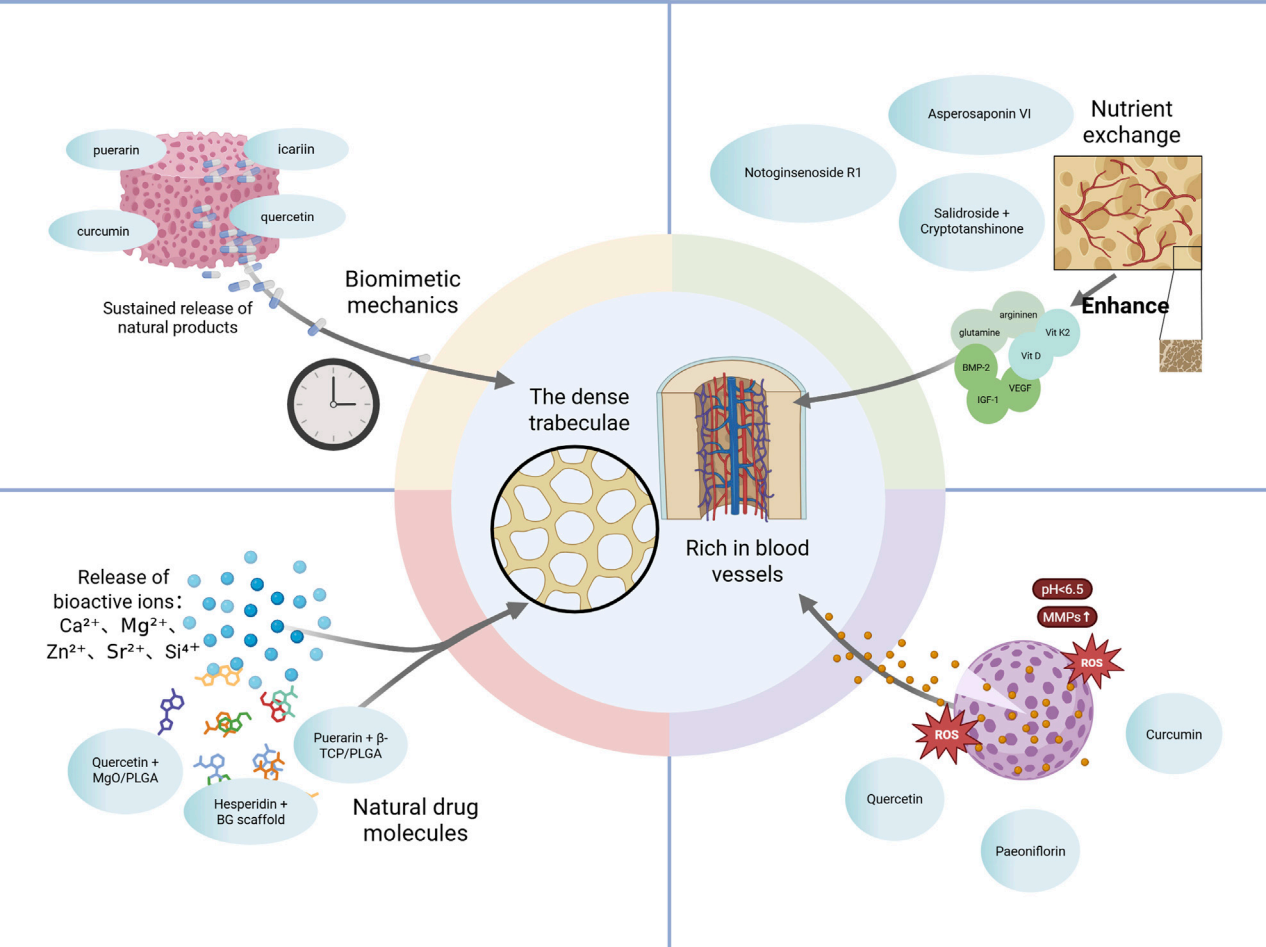
Synergistic Mechanisms of Natural Products and Biomaterials in Promoting Bone Regeneration. Natural product-loaded biomaterials facilitate sustained and responsive release under acidic or enzymatic conditions. Released bioactive ions (e.g., Ca^2+^, Si^4+^) and phytochemicals promote osteogenesis and angiogenesis. Surface nano-topographies and biomimetic mechanical properties enhance cell infiltration and nutrient exchange. Together, these integrated strategies support regeneration of vascularized bone within dense trabecular regions. Abbreviations: β-TCP, beta-tricalcium phosphate; PLGA, poly (lactic-co-glycolic acid); BG, bioactive glass; ROS, reactive oxygen species; MMPs, matrix metalloproteinases; pH < 6.5, acidic microenvironment (pH lower than 6.5); BMP2, bone morphogenetic protein 2; VEGF, vascular endothelial growth factor; IGF-1, insulin-like growth factor 1; Vit K2, vitamin K2; Vit D, vitamin D.

Recent studies have reported that the integration of natural compounds such as puerarin, curcumin, and hesperidin, a flavanone glycoside extracted from the peels of *Citrus aurantium* L. (Rutaceae), into engineered delivery systems—including PLGA/β-TCP composite scaffolds, DNA tetrahedron nanostructures, and BG matrices—potentiates their osteogenic and angiogenic activities in OP-related animal models (Li Y et al., 2024; Saberian et al., 2024; Wang et al., 2025). These findings suggest that the biomaterial-assisted delivery of plant-derived bioactives may offer a translationally viable route to overcome current limitations and enhance bone repair in metabolic bone disorders. These findings collectively demonstrate the synergistic potential of natural products and biomaterials in enhancing bone regeneration through controlled release, bioactive ion release, surface functionalization, and responsive delivery systems (Figure 6; Table 3).

**TABLE 3:** Bioactive product-loaded scaffolds and their applications in preclinical bone defect models.

Material type	Compound	Botanical source	Release profile	Animal model	Main outcome	References
Bioglass–carboxymethyl chitosan–silk fibroin (BG-CMC-SF) composite scaffold	Naringin	a flavanone glycoside isolated from the peels of *Citrus paradisi* Macfad. and *Citrus grandis* (L.) Osbeck (Rutaceae)	Sustained release over 15 days (80% cumulative release by day 15)	Rat calvarial critical-size defect model	↑Collagen, BV/TV, Tb.N; enhanced bone repair	Z. Wang et al. (2025)
Coaxial electrospun nanofiber membrane composed of polycaprolactone (PCL) and gelatin	Salidroside	a phenylpropanoid glycoside isolated from the roots of *Rhodiola rosea* L. (Crassulaceae)	Biphasic release pattern: initial burst release within 72 h followed by sustained release over 14 days	Rat femoral condyle bone defect model (SD rats)	↑Runx2, OCN, CD31; enhanced bone and vessel repair	Wu et al. (2024)
	Cryptotanshinone	a diterpenoid quinone isolated from the roots of *Salvia miltiorrhiza* Bunge (Lamiaceae)				
PLGA/MgO/Quercetin nanofiber membrane (nano artificial periosteum)	Quercetin	a flavonol widely distributed in the flowers of *Sophora japonica* L. (Fabaceae), the bulbs of *Allium cepa* L. (Amaryllidaceae), and the leaves of *Camellia sinensis* (L.) Kuntze (Theaceae)	Sustained release over 7 days (dual release of Mg^2+^ and quercetin)	Rat calvarial defect model (critical-size defect)	↑Runx2, ↑OCN, ↑CD31, Bone and blood vessel repair via Wnt/β-catenin activation	He et al. (2022)
PLGA/β-TCP composite scaffold	Puerarin	an isoflavone glycoside isolated from the roots of Pueraria lobata (Willd.) Ohwi (Fabaceae)	Sustained release over 21 days	Critical-sized calvarial bone defect model (SD rats)	↑Runx2, ↑OCN, ↑CD31, Bone and blood vessel repair	Cao et al. (2022)
PLLA scaffold	Panax notoginseng saponins	a mixture of dammarane-type triterpenoid saponins extracted from the roots of *Panax notoginseng* (Burk.) F.H. Chen (Araliaceae), combined with Osteopractic total flavone (OTF), a total flavonoid fraction isolated from the aerial parts of *Epimedium brevicornum* Maxim. (Berberidaceae)	Sequential release (PNS first, then OTF)	GC-induced osteonecrosis of femoral head (ONFH) in rats	↑Runx2, ↑OCN, ↑CD31, Bone and blood vessel repair	Feng et al. (2023)
Polydopamine (PDA)-coated titanium implant	Salidroside	a phenylpropanoid glycoside isolated from the roots of *Rhodiola rosea* L. (Crassulaceae)	Sustained release of salidroside over 7 days	SD rats with femoral implant surgery (osseointegration model)	↑Runx2, ↑OCN, ↑CD31; improved bone formation and vascularization around implants	Yi et al. (2022)
Polyurethane/nano-hydroxyapatite (PU:n-HA) composite scaffold	Gastrodin	a phenolic glucoside isolated from the tubers of *Gastrodia elata* Blume (Orchidaceae)	Sequential release: initial burst followed by sustained release over time	Rat femoral defect model	↑Runx2, ↑OCN, ↑CD31; bone and vessel repair	Li et al. (2023)
Silk fibroin/micro-nano hydroxyapatite/sodium alginate (SF/m-nHA/SA) composite scaffold	Ginsenoside Rb1	a dammarane-type triterpenoid saponin isolated from the roots of *Panax ginseng* C.A. Mey. (Araliaceae)	Sustained release over 16 days (initial burst followed by slow release)	Critical-size calvarial defect in SD rats	↑Runx2, ↑OCN, ↑BV/TV; promoted bone regeneration	Wu et al. (2022)

### 5.1 Controlled release strategies in bioactive scaffolds

Incorporating natural bioactive products into biodegradable polymer–ceramic composite scaffolds represents a promising strategy in bone tissue engineering, enabling localized, sustained, and controllable drug release at bone defect sites. Compared to conventional systemic or local injection approaches, these scaffolds not only enhance drug accumulation and retention within the defect region but also synergize with the intrinsic osteoinductive and pro-angiogenic properties of the carrier materials to optimize the bone regenerative microenvironment.

For instance, Cao et al. developed a PLGA/β-TCP composite scaffold loaded with puerarin, which exhibited sustained *in vitro* release for up to 5 months. *In vivo* studies demonstrated that this delivery system significantly promoted both osteogenesis and neovascularization, as evidenced by increased expression of VEGF and BMP-2, indicating its potential applicability for long-term bone regeneration in osteoporotic conditions (Cao et al., 2022). PLGA is well known for its excellent biodegradability and tunable release kinetics, making it particularly suitable for the delivery of poorly water-soluble phytochemicals such as puerarin. In contrast, β-TCP—a bioresorbable ceramic with superior osteoconductivity—facilitates bone integration and is particularly advantageous in chronic bone defect models.

Additionally, Gong et al. designed a coaxial electrospun polycaprolactone (PCL)/gelatin nanofiber membrane co-loaded with salidroside, a phenylpropanoid glycoside isolated from the roots of *Rhodiola rosea* L. (Crassulaceae), and cryptotanshinone, a diterpenoid quinone extracted from the roots of *Salvia miltiorrhiza* Bunge (Lamiaceae), which enhanced both osteoblastogenesis and angiogenesis via activation of the Wnt/β-catenin signaling pathway in a murine calvarial defect model (Gong et al., 2018). This coaxial nanofiber structure, which mimics the native periosteum, provides a high specific surface area, favorable cellular adhesion, and the ability to deliver dual agents in a spatially controlled manner—making it particularly suitable for membrane-guided regeneration in shallow or non-load-bearing bone defects.

Similarly, He et al. developed an electrospun “artificial periosteum” membrane composed of PLGA, magnesium oxide (MgO), and quercetin. This scaffold simultaneously promoted osteogenic differentiation of BMSCs and tube formation by endothelial progenitor cells. The optimal formulation (20% MgO and 0.1% quercetin) exerted its pro-regenerative effects primarily via Wnt/β-catenin pathway activation (He et al., 2022).

From a drug delivery platform perspective, microspheres and nanoparticles offer customizable release kinetics and high drug-loading capacity; however, their release profiles can be influenced by carrier stability and are prone to initial burst release. In contrast, injectable hydrogels provide superior biocompatibility and spatial conformity, making them suitable for filling irregularly shaped defects. Nevertheless, their release precision is often compromised by environmental pH and enzymatic degradation. Electrospun membranes and Three-dimensional (3D) -printed scaffolds offer robust mechanical support and allow for spatially organized drug distribution, rendering them ideal for load-bearing or complex craniofacial defect repairs. Ultimately, platform selection should be guided by key considerations such as biodegradability, mechanical integrity, drug-release stability, and compatibility with specific disease models.

### 5.2 Functional synergy between BG and phytomedicines

BG represents a class of inorganic biomaterials characterized by high surface reactivity and the ability to release therapeutic ions (e.g., Ca^2+^, Si^4+^) upon contact with physiological fluids. These ions induce the formation of hydroxyapatite-like layers on the BG surface, thereby conferring strong osteoconductivity and promoting angiogenesis. Additionally, the mesoporous structure of BG provides an efficient drug-loading matrix, enabling the co-delivery of bioactive phytochemicals and inorganic ions in a synergistic manner.

For example, Wang et al. fabricated a porous scaffold composed of BG, carboxymethyl chitosan, and silk fibroin, which was further loaded with hesperidin. This multifunctional system facilitated the concurrent release of therapeutic ions and phytomedicine, thereby inducing hemostatic activity, immunomodulation via M2 macrophage polarization, neurovascular regeneration, and osteogenesis (Wang et al., 2025). In an osteoporotic cranial defect model, the scaffold significantly improved both bone volume and vascular density, suggesting its potential as a comprehensive therapeutic platform for complex bone defects.

At the material selection level, BG is widely considered an ideal carrier for natural product delivery due to its compositional similarity to native bone minerals, high surface bioactivity, and ease of surface functionalization. These features make BG particularly suitable for treating bone disorders associated with complex pathophysiological conditions such as OP or diabetic osteopathy, which typically involve oxidative stress, inflammatory dysregulation, and impaired angiogenesis. In biological environments, BG-derived ions such as Si^4+^ and Ca^2+^ not only promote apatite mineralization and support osteoblastogenesis but also regulate macrophage polarization and upregulate VEGF expression, thereby enhancing the local antioxidant and pro-angiogenic milieu. These effects complement the pharmacological actions of natural antioxidants such as hesperidin, quercetin, and paeoniflorin, a monoterpene glycoside isolated from the roots of *P. lactiflora* Pall. (Paeoniaceae).

In comparison to conventional drug carriers such as hydrogels or polylactic acid scaffolds, BG offers several distinct advantages:

1. Excellent mechanical strength and intrinsic osteoconductivity, allowing for use in load-bearing sites such as cranial or long bone defects;
2. Dual-release capability of inorganic ions and phytochemicals, supporting multimodal intervention in osteogenesis, angiogenesis, and immune modulation;
3. Intrinsic pH-buffering capacity and antibacterial activity, which are advantageous for mitigating postoperative inflammation and infection.

Nevertheless, the clinical translation of BG-based scaffolds is challenged by several limitations, including an uncontrollable degradation rate, insufficient toughness, and complex shaping processes. To address these issues, current research trends favor the combination of BG with natural polymers—such as gelatin, silk fibroin, or chitosan—to improve mechanical integrity, biocompatibility, and drug-release kinetics. These hybrid strategies broaden the applicability of BG-based systems for the treatment of diverse pathological bone conditions.

### 5.3 Stimuli-responsive and smart drug delivery systems

Under osteoporotic conditions, characterized by chronic inflammation, oxidative stress, and microenvironmental dysregulation, the development of stimuli-responsive and smart drug delivery systems offers promising strategies to enhance the *in vivo* bioavailability, stability, and targeting precision of natural products. These platforms enable context-specific release of bioactive agents, thereby improving the therapeutic precision of mechanistic interventions in bone regeneration.

For instance, Li et al. constructed a curcumin delivery platform based on a DNA tetrahedron nanostructure (tFNA), which significantly improved its physiological stability and cellular internalization efficiency. This system activated the NRF2/GPX4 signaling axis, leading to inhibition of ferroptosis, restoration of mitochondrial function, and enhancement of osteoblastogenesis in BMSCs. In a diabetic OP model, the tFNA–curcumin system exhibited substantial osteoprotective effects, suggesting its translational potential for redox-sensitive bone disorders (Li C et al., 2024).

In another study, Tan et al. designed a Gelatin methacryloyl (GelMA)/nano-hydroxyapatite (nHA) hydrogel incorporating notoginsenoside R1, a dammarane-type triterpenoid saponin isolated from the roots of *P. notoginseng* (Burk.) F.H. Chen (Araliaceae), which provided localized and sustained release of the bioactive product. This hydrogel system activated the Notch1/Akt signaling pathway, thereby promoting both angiogenesis and osteoblastogenesis in calvarial bone defects. The minimally invasive nature of this approach, combined with its structural repair capability, underscores its clinical applicability in craniofacial bone regeneration (Tan et al., 2025).

Furthermore, a composite system comprising asperosaponin VI (ASP VI), a triterpenoid saponin isolated from the roots of *Dipsacus asper* Wall. ex Henry (Caprifoliaceae), together with chondroitin sulfate and scaffold-immobilized BMP-2 exemplifies the potential of multi-target synergistic delivery strategies. ASP VI upregulates osteogenic markers such as *ALP* and *RUNX2* in BMSCs, while concurrently downregulating RANKL, thereby suppressing osteoclastogenesis. In combination with BMP-2, this system co-activates multiple regenerative signaling pathways, including the SMADs, TGF-β1, Vascular endothelial growth factor A (VEGFA), and OPG/RANKL axes. This tri-modal synergy between the bioactive product, scaffold material, and molecular pathways highlights a rational design framework for enhancing osteo-angiogenic coupling in smart delivery systems.

### 5.4 Amplification of natural product effects via material composition and structural design

Recent research highlights that the microarchitectural features of scaffold materials—such as pore size, channel interconnectivity, and surface topography—play a pivotal role in amplifying the synergistic effects between natural bioactive products and biomaterial platforms. Hierarchically organized porosity and interconnected microchannels facilitate cellular infiltration and neovascularization, while engineered micro- and nanoscale surface patterns enhance cellular adhesion, proliferation, and osteoblastogenesis.

For instance, Cho et al. fabricated a PCL/nano-hydroxyapatite scaffold using combined salt-leaching and 3D printing techniques, achieving an average pore size of 26 μm and a porosity of 42%. Subsequent modification with hydrogel-induced wrinkled surface micropatterns significantly improved the scaffold’s biocompatibility and bioactivity, as evidenced by enhanced cell adhesion and proliferation (Cho et al., 2021).

From a disease-adaptive design perspective, different pathological bone conditions demand tailored microenvironmental modulation strategies. For example, osteoporotic and senile bone defects are typically characterized by reduced vascularization and impaired bone marrow niches, requiring scaffolds that provide long-term mechanical support and pro-angiogenic activity. In contrast, diabetic bone disease and infectious bone defects present with chronic inflammation and microbial colonization, necessitating immunomodulatory, pH-buffering, and antibacterial functionalities to regulate local immune responses and mitigate infection. Consequently, scaffold design should account not only for physicochemical compatibility with drug properties (e.g., surface charge, hydrophilicity, degradation kinetics), but also for structural adaptations tailored to the specific demands of the pathological microenvironment.

Zhang et al. further emphasized that integrating plant-derived bioactive products with orthogonal regenerative products—such as scaffolds, growth factors, and seed cells—enables localized co-delivery that synergistically enhances both the mechanical integrity and biological performance of the scaffold. This strategy provides a translational framework for designing multifunctional therapeutic systems for complex bone regeneration (Zhang et al., 2020).

In terms of platform comparison, porous scaffolds offer superior osteoconductivity and tissue integration, making them suitable for repairing high-load-bearing defects. Hydrogel-based systems are advantageous for minimally invasive applications, injectable formulations, and soft tissue interface repair. Nanoparticle platforms, due to their high surface-to-volume ratio and tunable surface characteristics, excel in targeted delivery and pharmacokinetic modulation, particularly for unstable or poorly water-soluble natural products. Strategic integration of these delivery platforms should be guided by the chemical properties of the bioactive product, the target tissue environment, and therapeutic objectives to maximize efficacy and optimize biomaterial performance.

In summary, diversified platform design strategies—including controlled-release scaffolds, BG composites, stimuli-responsive nanocarriers, and structure-driven functional enhancements—offer substantial advantages in sustaining drug release, remodeling pathological microenvironments, and promoting osteo-angiogenic coupling. The interplay between material properties, pathological context, and drug mechanisms is a key determinant of regenerative efficacy. Future research should focus on rationally optimizing platform combinations and elucidating multi-pathway synergies to improve precision bone repair under complex disease conditions.

## 6 Discussion and perspectives

This review categorizes anti-osteoporotic natural products into five main groups: flavonoids, glycosides/saponins, phenolics, polysaccharides, and terpenoids. These products exert key biological effects through: Activating osteogenic and angiogenic pathways, such as Wnt/β-catenin, BMP/Smad, PI3K/Akt, MAPK, and eNOS, which upregulate *RUNX2*, *Osterix*, *ALP*, *COL1A1*, and *OCN*;Modulating immune responses by promoting M2 macrophage polarization, enhancing Treg function, and inhibiting NF-κB and JAK/STAT signaling, thereby restoring osteoimmune balance; Reducing oxidative stress, mainly via the NRF2/HO-1 and NRF2/GPX4 axes, which protect against ROS accumulation and ferroptosis; Engaging dual signaling pathways, especially in polysaccharides and flavonoids, that simultaneously activate BMP and Wnt cascades to enhance osteoinductive effects.However, most existing studies focus on the effects of individual products, while the interactive dynamics of signaling pathways under multi-product interventions remain poorly explored. Given that many phytochemicals act on overlapping or converging molecular targets (e.g., PI3K/Akt, BMP2, Wnt), potential synergistic or antagonistic crosstalk may significantly influence the osteo–angiogenic coupling process. Future research should systematically investigate these interactions to inform the rational design of combination therapies or polyherbal formulations.

Additionally, while this review primarily focuses on the mechanisms through which natural products regulate osteogenesis–angiogenesis coupling, it is important to note that gut hormones such as GLP-1 and GIP may also play critical roles in bone homeostasis. Although no direct experimental evidence currently exists demonstrating that natural products regulate bone remodeling via modulation of gut hormone secretion, previous studies have shown that GLP-1 receptor agonists can significantly increase bone mass, improve bone microarchitecture, and reduce bone resorption markers such as C-terminal telopeptide of type I collagen (CTX-1) in osteoporotic or diabetic models (Zhao et al., 2017; Cheng et al., 2022). These findings suggest that the “gut microbiota–gut hormone–bone” axis may represent a novel regulatory pathway worth exploring. Future research should investigate whether specific natural products can enhance gut hormone signaling or secretion—either directly or via microbiota modulation—as a means to indirectly promote osteogenesis and inhibit osteoclastogenesis. Integrating this dimension could enable more systemic therapeutic strategies and broaden the translational scope of natural product–based interventions in bone metabolic disorders.

Despite promising preclinical outcomes, clinical translation faces several major challenges. Most current animal studies rely on traditional OVX rodent models or monoculture cellular assays, which, although informative for bone loss mechanisms, are insufficient for recapitulating the complex bone–vascular–immune interactions seen in humans. More disease-relevant models—such as type 1 diabetes (T1DM)-induced bone defects or microbiota-perturbed systems—have been explored but remain limited in their capacity to simulate systemic crosstalk. Thus, advanced experimental models, including 3D co-culture systems, organ-on-a-chip platforms, and humanized mouse models, should be prioritized to enhance the physiological relevance and translational potential of studies evaluating natural metabolite interventions.

Furthermore, the clinical application of natural products is hindered by insufficient standardization, unclear pharmacokinetics, and limited delivery strategies. Many extracts or multi-component formulations lack consistency in batch production, quantitative characterization of bioactives, and robust quality control, thereby limiting reproducibility and regulatory compliance. In addition, oral and localized administration routes differ significantly in tissue exposure, pharmacokinetics/pharmacodynamics, and safety margins—yet comprehensive studies on dosage optimization, tissue targeting, and half-life remain lacking. Long-term safety assessments and evaluations of combinability with existing osteoanabolic or anti-resorptive therapies are also critically needed. From a materials science perspective, the manufacturability, post-sterilization stability, and scalability of delivery platforms must be further optimized for clinical deployment.

To address these challenges, we propose a translational roadmap centered on three core pillars: elucidation of mechanisms, optimization of dosing strategies, and development of intelligent delivery systems. Cross-scale validation of key signaling hubs—such as VEGF/Notch/eNOS, Wnt, and NRF2/GPX4—should be conducted across cellular, small animal, and large animal models to establish a robust body of evidence. Harmonization of outcome metrics—including CD31^hiEmcn^hi vessel density, bone volume fraction (BV/TV), BMD, and mechanical strength—will enhance comparability and clinical relevance. Early-phase clinical translation should focus on localized scenarios with high feasibility, such as bone defect repair, peri-implant bone preservation, and alveolar ridge maintenance.

To accelerate the clinical translation of natural bioactives for osteoporosis, we recommend the following research priorities: (1) Standardize extraction processes and establish methods for component identification and quantification; (2) Focus on well-characterized, multi-target candidates such as icariin, curcumin, and ginsenoside Rb1; (3) Customize delivery systems based on patient-specific variables (e.g., sex, age, metabolic status, and defect morphology) and integrate stimuli-responsive elements (e.g., pH- or enzyme-triggered release) for improved temporal control; (4) Develop clinically relevant animal models, such as postmenopausal or diabetic osteoporosis, and unify outcome evaluation criteria; (5) Based on mechanistic strength and delivery compatibility, “ICA + PLGA scaffold” and “curcumin + tFNA nanodelivery system” are proposed as the most promising translational candidates.

Building upon the current mechanistic insights, natural products interacting with the bone–vascular–immune microenvironment, particularly through biomaterial-facilitated delivery, offer a promising foundation for next-generation therapies targeting bone degenerative diseases. To facilitate clinical translation, future research should address current limitations in pharmacological standardization and deepen our understanding of their genetic and epigenetic modes of action, including regulation of non-coding Ribonucleic acids (RNAs) and chromatin remodeling.

## 7 Conclusion

Natural bioactive products from botanical sources provide a multi-target and mechanistically diverse strategy for preventing and treating osteoporosis and related skeletal disorders. These compounds directly regulate key signaling pathways—such as Wnt/β-catenin, BMP/Smad, PI3K/Akt, MAPK, and RANKL/NF-κB—to promote osteogenesis and suppress osteoclastogenesis. Beyond cellular regulation, they reshape the bone microenvironment by enhancing angiogenic coupling (VEGF/Notch/eNOS), modulating immune balance through M2 macrophage polarization and Treg induction, and alleviating oxidative stress or ferroptosis via the Nrf2/GPX4 axis. The gut microbiota–SCFA–bone pathway further extends their systemic influence on bone remodeling. Incorporating these phytochemicals into advanced biomaterial-based delivery systems, including PLGA, β-TCP, and bioactive glass scaffolds, enhances bioavailability, stability, and controlled release. Collectively, the integration of natural products with functional biomaterials provides a translationally promising framework for bone regeneration, paving the way toward standardized, smart, and clinically applicable therapies for metabolic bone diseases.
